# Evidence of the contribution of molecular fluorophores to the luminescence of carbon entities formed by solvothermal treatment of trinitropyrene†

**DOI:** 10.1039/d4ra07553f

**Published:** 2024-12-19

**Authors:** Rayan Roch, Xavier Deschanels, Chandra Mohan Singaravelu, Noé André, Cyrielle Rey, Jérémy Causse

**Affiliations:** a ICSM, University Montpellier, CEA, CNRS, ENSCM 30207 Marcoule France rayan.roch@gmail.com xavier.deschanels@cea.fr jeremy.causse@cea.fr

## Abstract

Carbon dots are a subset of carbon nanomaterials with fluorescent properties that render them attractive for various potential applications such as bioimaging and sensing. The past years saw significant progress being made in the understanding of the formation and the underlying fluorescent property. Nevertheless, efforts are still necessary to unravel the formation of carbon dots and the origin of their luminescence, especially for new types of precursor material such as polycyclic aromatic compounds. Trinitropyrene, a nitroaromatic derivative of pyrene, is increasingly being used as an organic precursor for carbon dot synthesis by bottom-up method. This work aims to study the luminescent products obtained by microwave-assisted solvothermal treatment of trinitropyrene in a common organic solvent for nanoparticle synthesis, dimethylformamide. By employing flash chromatography, we isolated different fractions from which mainly stems the fluorescence observed from the crude sample obtained post solvothermal treatment. By performing structural and spectroscopic characterization techniques, we observed that they possess quiet similar chemical composition and luminescent properties but significant differences from a structural point of view. From these observations, we suggest that the fractions mainly consist of molecular derivatives of the precursor material. This study calls attention to the need of separation and purification techniques in order to better assess the properties of carbon dots.

## Abstract 2

Carbon dots are a subset of carbon nanomaterials with fluorescent properties that render them attractive for various potential applications such as bioimaging and sensing.

## Graphical abstract

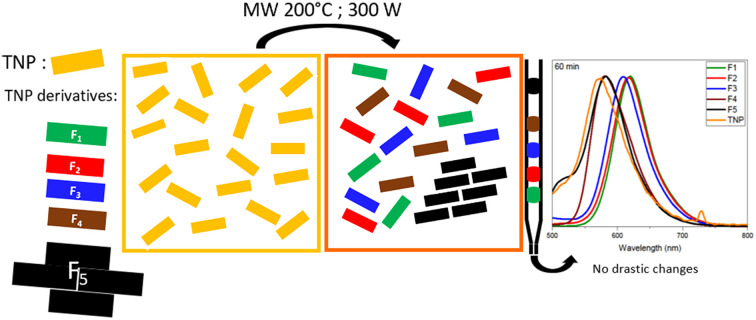

## Introduction

Carbon dots (CDs) are a subset of fluorescent carbon nanomaterials serendipitously discovered in 2004 during the purification of single-walled carbon nanotubes.^1^ They display remarkable physicochemical properties such as photoluminescence, solubility in aqueous and/or organic media,^2–4^ and photostability as well as potential fields of application like bioimaging,^5,6^ sensing^7–9^ and optoelectronics.^10–12^ There is a growing interest in these nanoparticles due to the simplicity of their synthesis protocol and the accessibility of precursors, leading to the publication of many articles in recent years. Carbon dots are defined by a characteristic size of less than 10 nm and a quasi-spherical shape. These nanoparticles consist of a predominantly carbon core with amorphous and crystalline areas along with chemical groups on the surface. Carbon dot synthesis approaches can be separated in two categories: bottom-up and top-down synthesis. In the top-down approach, other carbon allotropes are fragmented to attain the desired nanoparticles through laser ablation, arc discharge, electrochemical,^13–15^ and acidic treatment. In the bottom-up approach, the nanoparticles are built from small organic molecules that undergo treatments such pyrolysis, microwave, and solvothermal treatments. Bottom-up synthesis of carbon dots has seen great progress in particular. Most of the attention is directed on citric acid systems and a few aromatic compounds such as phenylene diamine and phenolic derivatives.^16^ However, recent works highlighted the formation of fluorophores of citrazinic acid derivatives in citric acid systems. Moreover, methods of purification and separation are often insufficient or inadequate, if any at all. Formation of molecular fluorophores and inadequate purification methods leads to properties misattributed to carbon dots and is detrimental for the better understanding of these nanoparticles. Polycyclic aromatic compounds are increasingly used as precursors for bottom-up synthesis. They possess large conjugation domains, an attractive feature for some optical properties such as NIR fluorescence.^17^ Trinitropyrene (TNP) is a reactive pyrene derivative that is being growingly reported in recent years as a precursor for CDs synthesis.^18–29^ Wang *et al.* reported the first synthesis of carbon dots involving trinitropyrene as the precursor.^18^ Graphene quantum dots (GQDs) with remarkable crystallinity and optical properties were described in the literature, as a type of CDs composed of one or a few nanosized graphene layers stacked that display well-ordered morphology. Wang and his coworkers presented a room temperature synthesis of GQDs *via* electron-beam radiation and a demonstration of its application as a fluorescent probe for cell imaging. Wu *et al.* reported organic soluble carbon dots with high mass yield.^20^ They display long wavelength emissions and high solubility in a wide range of polar and non-polar solvents. Trinitropyrene was also employed jointly with various co-precursors such as boric acid,^24,25,27,29^ polyethylene-imine,^23^ 2,4-diaminobenzenesulfonic acid,^28^ diethylenetriamine^26^ and in different solvents.^21^ However, despite of the increasing numbers of articles employing trinitropyrene as a precursor, studies on the structural and emissive properties of TNP-derived C-Dots were only recently addressed. Batra *et al.* investigated three fluorescent components in CDs from TNP.^30^ Using a series of separation techniques, they showed that the sample was constituted with components with different weight percentages in the synthesis mixture and different quantum yield (QY). The isolated components presented distinct chemical and optical properties. They suggested that the fraction with the highest QY and mainly responsible for the observed emission corresponds to molecular fluorophore in the form of pyrene derivatives. They also came to the conclusion that the fraction with the second highest QY displays a quasi-CNDs like structure and the fraction with the lowest QY is the one the exhibiting coherent structural properties with what is expected from CDs. CNDs are defined as a type of CDs with a varying proportion of amorphous and ordered carbon in a sphere-like shape. Kothalawala *et al.* studied the nature and properties emissive components produced through hydrothermal treatment of TNP.^31^ They isolated small molecular fluorophores with clearly different chemical structure and optical properties significantly brighter emission than the CDs obtained in the same synthesis. Batra and Kothalawala both showed that the constituents of the mixture obtained after hydrothermal treatment were of different nature and showed distinct optical properties compared to the CDs produced simultaneously/alongside. Most of them displayed significantly brighter emission than CDs. Nonetheless, they both worked in specific hydrothermal conditions. Even if numerous reports of synthesis based on trinitropyrene in organic solvents have been made, the synthesis was never investigated in organic medium to such an extent to our knowledge. Hence, this study is focused on the solvothermal treatment of TNP in dimethylformamide (DMF), a commonly used organic solvent used in solvothermal synthesis, in order to investigate the nature and properties of the fluorescent constituent obtained in these conditions.

## Experimental

### Materials and methods

All chemicals and solvents used were purchased from commercial suppliers and used without further purification as follows. Nitric acid (ACS reagent, 69%), sulfuric acid (RPE, 96%), *N*,*N*-dimethylformamide (ACS reagent, ≥99.8%), ethanol (RE grade), cyclohexane (RPE grade), dichloromethane (RE grade), methanol (RE grade), NaOH were purchased from Carlo Erba. Pyrene was purchased. Milli-Q water was used during the experiment. Flash chromatography was performed on a Reveleris X2 flash chromatography system from BUCHI using 25 g Flashpure Ecoflex column containing 50 μm irregular silica. Dialysis membranes (regenerated cellulose, 1 kDa MWCO) were purchased from Spectra/Por.

### Synthetic procedures

#### Solvothermal treatment of TNP

The procedure to obtain trinitropyrene was based on a method proposed in the litterature^20^ In a 500 ml-round bottom flask, 60 ml of HNO_3_ and 20 ml of H_2_SO_4_ were mixed. 1 g of pyrene was added and the mixture was heated at 80 °C under stirring (750 rpm) for 48 hours. The flask was opened twice a day in order to eliminate the reddish fumes generated during the synthesis. After cooling to room temperature, 300 ml of water was slowly added. The mixture in flask was collected in centrifugation tubes. Following the centrifugation of the tubes, the supernatant was discarded, fresh water was added and the precipitate was redispersed. This operation was repeated until the pH of the supernatant after centrifugation was neutral. The resultant powder (1.44 g) was dried in an oven at 60 °C overnight and assumed to be TNP.

In a 35 ml vial containing 100 mg of TNP, 10 ml of DMF was added. The mixture was heated in a CEM Discover SP microwave system at 300 W and 200 °C under high stirring during determined duration (20 min, 40 min, 60 min). After cooling to room temperature, a solvent exchange was performed during 6 hours using a semi-permeable membrane (regenerated cellulose, 1 kDa) to replace DMF with EtOH. The dialysate was replaced every two hours. The solution containing the sample in the dialysis tube was retrieved for further purification. Centrifugation ultrafiltration was performed on the sample with a centrifugal filter (Vivaspin 15R, 2 kDa) at 4000 rpm at 35 °C during 1 h. The filtrate was discarded, ethanol was added to the filter device. The operation was repeated until the filtrate was almost clear. The sample was retrieved and dry-loaded on silica gel for further purification. Flash chromatography was performed through a Puriflash system with a binary solvent mixtures cyclohexane/dichloromethane, respectively solvent A and B. A linear gradient elution method was executed as follows: a volume ratio of 50% of B, hold it for 3 column volumes (CV); 50% to 100% of B over 10 CV; 100% of B, hold it for 3 CV. In an attempt to collect a part of the sample that was barely eluting, another step with 90 : 10 ratio of dichloromethane : methanol was added afterward, for 5 column volumes. Silica gel columns of 24 g were used as the stationary phase and equilibrated with 6 column volumes of 50% of each solvent. After collecting the tubes, the corresponding fractions were reassembled each in a round-bottom flask. The eluting mixture was removed using a rotary evaporator. Because the sample was spread in the flask, a small volume of acetone was added, the flask was rotated by hand in a sonicating in order to collect all the sample spread on the wall of the flask. The sample was then dried in an oven at 40 °C overnight.

### Characterization

Chemical structures and optical properties were obtained by X-ray diffraction analysis (XRD), Raman spectroscopy, Fourier transform infra-red spectroscopy (FTIR), UV-visible spectroscopy (UV-vis), High Resolution Mass Spectrometry (HRMS), Fluorescence spectroscopy, Scanning Electron Microscopy (SEM). UV-vis absorbance measurements of the fractions obtained by column chromatography in toluene were carried out using a Shimadzu UV-3600 spectrophotometer. The spectra were collected using a quartz cuvette with a 10 mm path length and a 3 ml volume. The samples were scanned in the wavelength range of 300–800 nm with a bandwidth of 3 nm and 1 s integration time. Emission spectra of the samples were recorded using a Horiba Scientific FluoroMax-4. The measurements were performed at a series of excitation wavelengths using a 2 nm entrance and 1 nm exit slits with an integration time of 0.1 s. 3D fluorescence mapping spectra were recorded in the excitation wavelength range of 250–600 nm and emission wavelength range of 550–700 nm. The value of the entrance and exit slit widths used were 1 nm for the column fractions and 2 nm for the TNP. Fourier transform Infrared (FTIR) spectra were recorded using a PerkinElmer IR-FT100 spectrometer equipped with an attenuated total reflection (ATR) module. The scans were recorded with a resolution 4 cm^−1^ of and 10 repeated scans. The samples were placed in powder form on the ATR plate. Raman spectra were measured using a confocal microscope Raman LabRAM Aramis spectrometer equipped with a 532 nm excitation. The samples were placed in powder form on a glass plate. Diffraction patterns were recorded on a Bruker D8 Advance diffractometer using a Cu Kα radiation source, from 5° to 70°. SEM images were taken with a FEI QUANTA 200 ESEM FEG microscope. HRMS was conducted using a Orbitrap ID-X (Waters) device and dichloromethane was used as the solvent. Samples were ionized following Electrospray Ionization technique (ESI).

The quantum yield values were measured with respect to the standard Fluorescein reference in 0.1 M NaOH using the slope method. The QY value of each sample was subsequently obtained using the following equation:
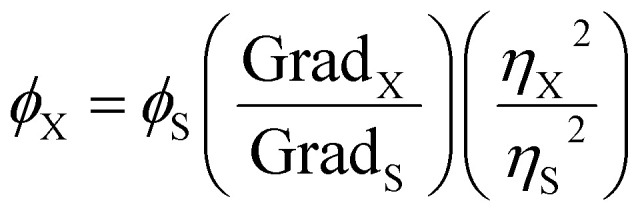
where the subscripts S and X denote standard and sample respectively, *Φ* is the fluorescence quantum yield, Grad is the gradient from the plot of integrated fluorescence intensity *vs.* absorbance, and *η* is the refractive index of the solvent.

## Results and discussion

### Solvothermal treatment of TNP

**Fig. 1:**
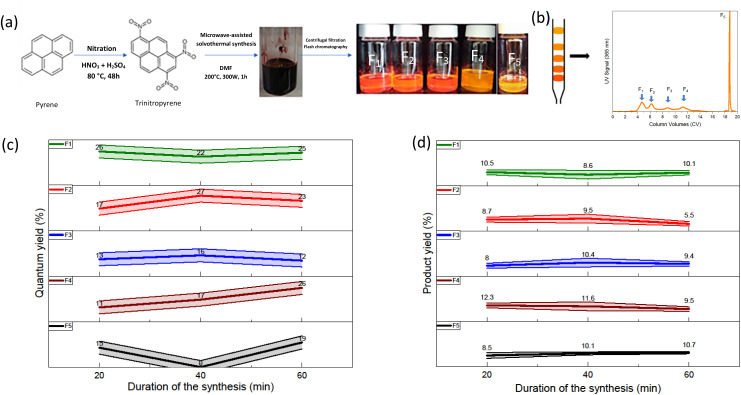
(a) Schematic of the microwave-assisted solvothermal treatment of trinitropyrene in dimethylformamide. (b) Chromatogram of a sample (c) relative quantum yield for a given fraction depending on the synthesis duration (d) product yield for a given fraction depending on the synthesis duration.

The solvothermal treatment of nitrated pyrene-derived CDs was done using a microwave synthesizer (Fig. 1(a)). TNP was heated with DMF as the solvent under pressure as described in the experimental procedure section. After solvothermal treatment, the mixture is black opaque with dark reddish highlights. A solvent exchange step was performed in order to replace DMF, a high boiling point solvent with ethanol. This method allows the removal of DMF without heating the mixture. Indeed, further reactions could occur because of additional heating. The low CDs solubility in ethanol and results in an only small quantity of the sample thrown away with the DMF-containing ethanol eliminated. CDs obtained from trinitropyrene have been reported to display good solubility in low polarity solvents.^21^ Ultrafiltration was performed in order to separate the products from smaller molecular weight impurities. The powder obtained was black and corresponded to a 90% mass yield for the synthesis. It displayed a low solubility in ethanol with a faint emission and good solubility in low polarity solvents such as toluene with an orange-red emission. In order to gain information on the sample obtained, silica column chromatography was executed (Fig. 1(b) and S.I.A†). Several fractions were obtained after this step labelled as F_1_, F_2_, F_3_, F_4_ and F_5_ according to their eluting order. The duration of retention increases from F_1_ to F_4_ and F_5_ eluting afterward in different elution conditions due to the fact that this fraction was kept in the stationary phase after the initial step of elution. Their different retention times are due to their interactions with the stationary phase, the silica. This stationary phase is a normal phase of general purpose which tends to bind highly polar compounds. The increasing retention times is attributed to an increasing polarizability of the fractions.

The solvothermal treatment was performed at three different durations (20 min, 40 min and 60 min) in order to investigate a possible kinetic effect on the products. The quantum yield values of all fractions are displayed in the Fig. 1(c). Overall, the fractions exhibit QY values around 20% taking into account the uncertainty of the relative quantum yield method. There is no specific trend in the evolution of the quantum yield depending on the duration of the synthesis. Depending on the eluting order, the first and second fraction seems to exhibit a slightly higher quantum yield compared to the other fraction. However, compared to what Batra *et al.* and Kothalawala *et al.* reported, the variation is hardly significant. We note first that we don't obtain the same number of fractions. Indeed, whereas they both obtained three main fractions, we obtained five fractions. Batra *et al.* and Kothalawala *et al.* obtained respectively QY values of 12% and 31% for their first eluting fraction, 8% and 23% for their second eluting fraction and 0.8% and 1% for their last eluting fraction. The product yield values of the fractions are also displayed in the Fig. 1(d). The product yield of the fractions is in general around each 10%. A significant part of the other half barely migrates during the column chromatography step and stays stuck to the silica stationary phase. Since no significant variations were observed regarding the quantum and mass yields, we will focus on the synthesis carried out at a duration of 60 min from hereon.

### Raman, FTIR, HRMS

To obtain more insight on the weight of the different fractions, high resolution mass spectrometry was performed (see S.I.B/†). Electrospray ionization-mass spectrometry was executed in both positive and negative mode. Overall, in positive mode, the spectra of the different fractions present a pseudo molecular ion with a similar *m*/*z* mass around 415–420. Several peaks are in common between the different fractions in addition to the molecular pseudo-ions. Some peaks correspond to monocharged adducts with different ions (Na^+^, NH_4_^+^, K^+^) commonly observed in positive mode ESI-MS spectra (see Table S.I.B/1 and Fig. S.I.B/2 from ESI†). Less intense peaks of lower or higher *m*/*z* values are attributed to multimers and multicharged adducts. The peaks at *m*/*z* 336 correspond to traces of TNP given that M(TNP) = 337 g mol^−1^. When the pseudo molecular ion [M + H]^+^ has the value *m*/*z* = 415, the peaks at 432 and 437 are attributed to the monocharged adducts [M + NH_4_]^+^ and [M + Na]^+^ respectively. The peaks at *m*/*z* = 846 are assigned [2M + NH_4_]. When the pseudo molecular ion [M + H]^+^ has the value *m*/*z* = 420, the peaks at 437 and 442 are attributed to the monocharged adducts [M + NH_4_]^+^ and [M + Na]^+^ respectively. The peaks at *m*/*z* = 856 are attributed to the multimer [2M + NH_4_]. The results obtained in negative mode are slightly different. The *m*/*z* values of the different fractions do not vary a lot between themselves with values around 352 to 382. Other notable intense peaks can be observed for *m*/*z* values of 309, 380 and even 410. We observed the fact that the values in positive mode are on average higher than in negative mode. We made the hypothesis that an adduct might form between derivatives of the precursor material and another compound such as dichloromethane or dimethylformamide and that it might be more easily detected in positive mode. It has to be noted that, in positive mode, if we subtract 84 (*ca.* the value of the molar mass of dichloromethane, the solvent used during the mass spectrometry analysis from the *m*/*z* values of the peaks considered to be originating from the pseudo molecular ion (*ca.* 415 or 420), we obtain values around 331 and 336. Moreover, in negative mode, subtracting 73 (*ca.* the value of the molar mass of dimethylformamide), from peaks with *m*/*z* values around 410, 426 or 442 yields values around 337, 353 and 369 which are close to more intense peaks detected. Peaks at around *m*/*z* = 337 were detected again. Overall, most of the *m*/*z* values detected vary between 279 and 420. This suggests that these fractions are mainly be composed of various nitropyrene derivatives.

**Fig. 2:**
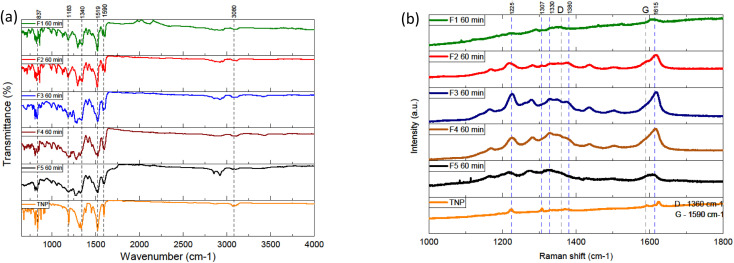
(a) FTIR spectra of the fractions and the trinitropyrene (b) Raman spectra of the fractions and trinitropyrene.

FTIR spectroscopy was performed in an attempt to better assess the chemical compositions of the samples (Fig. 2(a) and S.I.C/†). Overall, the spectra of different fractions display intense peaks in the 750–1600 cm^−1^ region and smaller peaks between 2800 and 3200 cm^−1^. The fractions have several overlapping peaks in common with the spectrum of TNP, which indicates similarities from a structural point of view. The intensities of the peaks are not significantly different for the main ones. The intense peaks around 1516 and 1346 are attributed to the asymmetric and symmetric stretching of the NO_2_ bond respectively. These intense peaks present in every FTIR spectra including the TNP, which suggest that the sample still contains significant nitro group. Additionally, the fractions show peaks corresponding to aromatic sp^3^ C–H stretching (3100), C–H stretching (2920 cm^−1^), C=C stretching (1590 cm^−1^) and NO_2_ shearing (860 cm^−1^). We notice the absence of broad intense hydroxyl peaks (around 3400 cm^−1^). In order to probe the chemical structure of the samples, the samples and precursor were analysed by Raman spectroscopy (Fig. 2(b) and S.I.D†). The acquisition of Raman spectra was negatively impacted by the intense fluorescence in the background. The Raman excitation laser causes damage to the sample because of the localized heat. Laser attenuators were used in order to diminish this effect. Raman spectra of carbon dots are expected to display two bands in particular: the D band (1360 cm^−1^) and the G band (1590 cm^−1^). They correspond respectively to the amorphous sp^3^ hybridized carbon and the ordered sp^2^ hybridized carbon in the material. Overall, the Raman spectra of the different fractions present intense large bands in common. Most of the intense ones are in common with TNP. The bands at 1590 cm^−1^ and 1279 cm^−1^ can be attributed to NO_2_ asymmetric stretching and symmetric stretching. The bands in the range 1000–1300 cm^−1^ are associated to CH in-plane bending vibrations. Overall, the FTIR results are consistent with the Raman results, a further indication that the fractions are close to TNP in terms of chemical groups.

### Structural analysis (XRD, SEM)

**Fig. 3:**
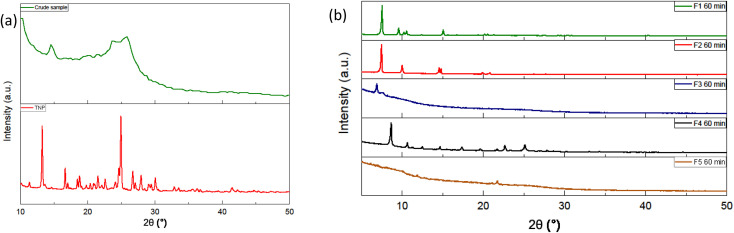
(a) Typical diffraction pattern obtained for the crude sample at the top and the diffraction pattern of the trinitropyrene (b) diffraction pattern of the fractions F_1_ to F_5_.

Before purification, the diffractogram of the raw samples exhibits a pattern similar to what is expected from carbon dots (Fig. 3(a)). A large peak around 25° is observed with no particular sharp peaks that could be attributed to a quantity of fluorophores or other by-products. However, the fractions obtained after purification display different diffraction pattern from the raw sample (Fig. 3(b)). The Fig. S.I.E.† contains the diffraction patterns of the fractions with and without the background. The fractions are also compared between themselves for a given duration or for a given fraction F_*n*_ (see ESI†). For a synthesis of 60 minutes, F_1_, F_2_ and F_4_ show sharp peaks between 5° and 40° and no large peak around 25° whereas F_3_ has a small sharp peak at 2*θ* < 10° and two less intense large peaks around 10 and 24°. The first fractions F_1_ eluting during the column chromatography displays very similar diffraction pattern. The main peaks are almost the same with the most intense one at 2*θ* ≈ 7.47°. These three samples also share common peaks among the less intense ones. It can be noted that the peak at 12° was attributed to plane {001} of pyrene and its derivatives.^31^ The diffraction pattern is very different from the raw sample showing that the fractions F_1_ present a more crystalline nature. The diffraction patterns of the second fractions F_2_ have a few common peaks but are not very similar. F_4_, in addition to the sharp peaks observed, displays underlying large peaks around less than 10° and 25° indicating the contribution of an amorphous phase. F_3_ also displays underlying large peaks around less than 10° and 25° but few sharp peaks. The diffraction pattern of F_5_ displays mainly quite large peaks around 10° and 20°. The fractions display really different diffraction pattern with the first ones exhibiting crystallinity and the last ones, especially F_5_, a rather amorphous phase.

**Fig. 4:**
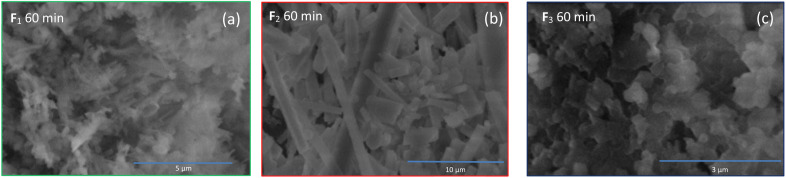
SEM micrographs of the fractions F_1_ (a), F_2_ (b) and F_3_ (c).

To perform in-depth structural characterization, scanning electron microscopy (SEM) and transmission electron microscopy (TEM) are presented in this section. Being organics samples, they charge up a lot under electron microscopy, which therefore produce white localized area. This is detrimental for the observations of the samples. Various aggregated structures were observed for the fractions obtained at the three different durations, 20 min, 40 min and 60 min. In this paragraph, we will only be discussing of the synthesis of 60 min duration (Fig. 4). The results for the 20 min and 40 min duration are available in the ESI† (see S.I.F/†). The TEM micrographs are also discussed in the ESI (see S.I.G/)† For the 60 min, the fraction F_1_ 60 min shows tangled, stacked thread-like structure of various size (μm size) and diameter The sample charge up a lot so the observation of the sample is difficult. Fraction F_2_ 60 min displays straight rods structure. F_3_ 60 min shows irregular sheets and sphere-shaped pieces. F_4_ 60 min and F_5_ 60 min doesn't display any particular morphology. F_1_ 60 min displays similarities to the F_1_ 20 min and F_1_ 40 min whereas F_2_ 60 min morphology is quite different from F_2_ 20 min and F_2_ 40 min. Overall, some fractions display quite regular structure. This imply that the sample structure is organized on a long range. This is in accordance with the XRD results that suggest that these samples are crystalline. This is not something expected for carbon dots but these morphologies are expected for organic molecules. Moreover, the ability of pyrene derivatives that self-assembled to form supramolecular object such as nanorods has been reported.^32–34^ This suggest, like the XRD and the HRMS results, that the samples are more likely not exactly CDs.

### Optical properties

**Fig. 5:**
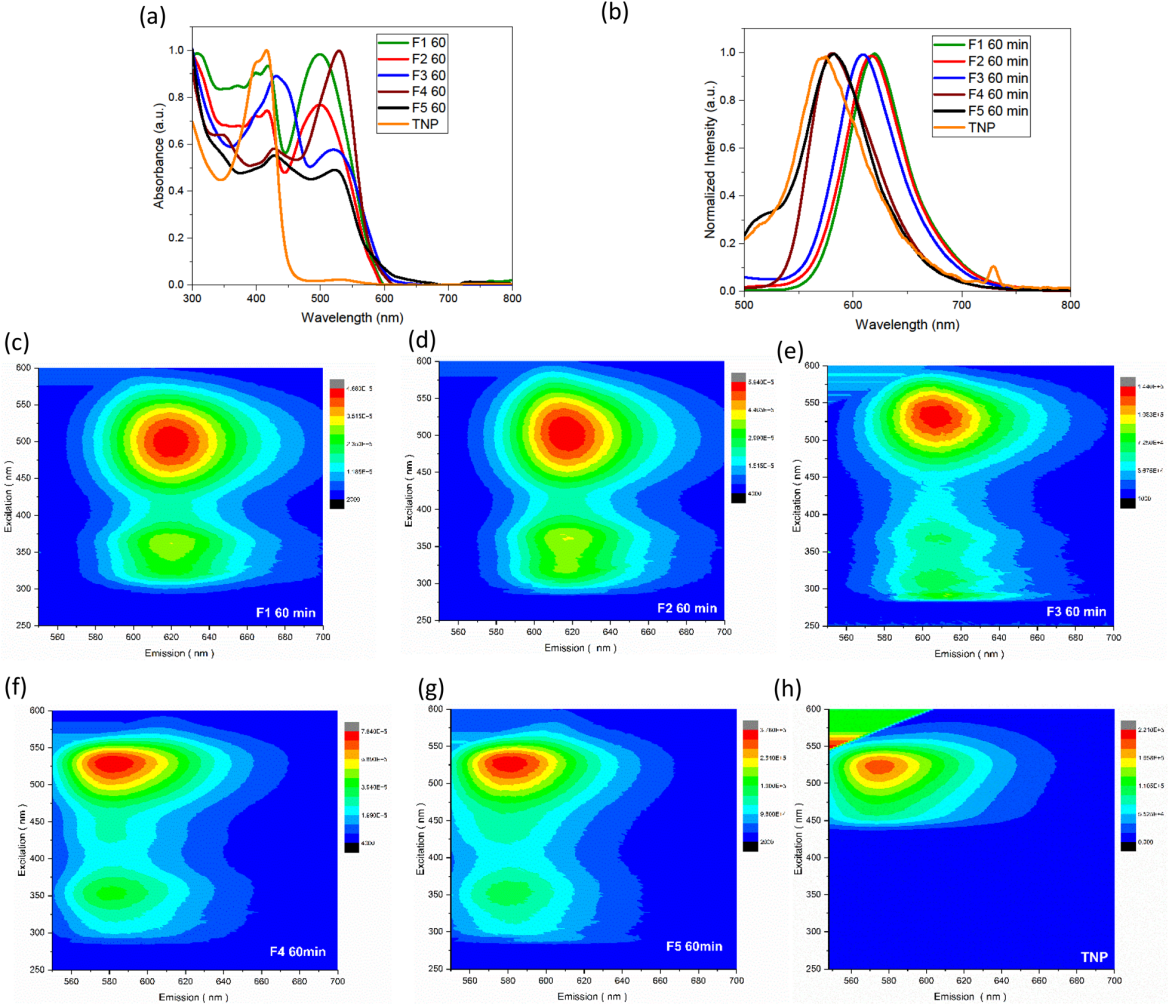
(a) UV-vis absorbance spectra of the 5 fractions obtained for a 60 minute duration solvothermal treatment and TNP (b) photoluminescence (*λ*_ex_ = 470 nm) spectra of the 5 fractions obtained for a 60 minute duration solvothermal treatment and TNP. 3-D photoluminescence mapping of F_1_ (c), F_2_ (d), F_3_ (e), F_4_ (f), F_5_ (g), and TNP (h) (coloured bars at the right display the photoluminescence counts per second).

The UV-vis spectra of the samples obtained after column chromatography and the trinitropyrene were recorded (Fig. 5). The fractions are also compared between themselves for a given duration or for a given fraction F_*n*_ (see Fig. S.I.H/1 and S.I.J/1†). For fraction F_1_ and F_2_, pronounced absorption is observed at around 400 and 500 nm. In the case of F_3_, its absorption bands are slightly shifted at 430 and 530 nm with the 430 nm band being significantly more intense than the 530. F_4_ and F_5_ displays similar absorption bands to F_3_. However, for F_4_, the 530 band is more intense than the 430 one and, for F_5_, both bands are of similar intensity. Around 400 nm and 500 nm, significative absorption bands are observed. F_4_ and F_5_ exhibit an absorption band around 360 nm. Except for these two fractions, there is not much absorption in the 300–400 nm area. Vibronic features and quite sharp bands can be observed. It contrasts with the expected large UV-vis absorption for carbon dots and is considered characteristic of molecular fluorophores.^35^ Moreover, the absorbance spectra quite closely resemble the absorption spectra of the trinitropyrene at the 400 nm region. Emission spectra was also recorded. The small peak around 730 nm for the TNP emission spectra is due to 2nd order scattering but doesn't change the overall fluorescence of the TNP spectra. From F_1_ to F_4_, brightly luminescence can be observed under 365 nm UV, lamp. The fraction F_5_ also shows luminescence alongside significant sedimentation at the bottom of the flask. Fractions F_1_ and F_2_ have the similar maximum wavelength emission of 618 nm. The maximum emission wavelength of F_3_ is slightly shifted to the shorter wavelength. F_4_ and F_5_ differ with a maximum wavelength emission of 580 nm. The 3D photoluminescence mapping of the fractions was also recorded. All the fractions exhibit excitation-independent behaviour. This suggests that the fractions are composed of one or more likely a few similar fluorophores.

## Discussion

The initial purpose of this experiment was to synthesize carbon dots during microwave-assisted solvothermal treatment of trinitropyrene. During this study, we observed that molecular fluorophores are formed. The diffractogram of the crude sample (Fig. 3(a)) obtained after the solvothermal treatment corresponds to what is expected from carbon dots. In order to better assess the composition of the sample, a chromatography separation was executed inspired from a method previously reported in the literature.^31^ After adding some adjustment to the procedure, we sorted the initial sample in five different fractions. Overall, the fractions obtained display similar photoluminescent properties but different structural properties (see Table S.I.K/†). After characterization, the first four eluted fractions are identified as molecular fluorophores. The last fraction eluted, F_5_, is strongly suggested as containing carbon entities, likely carbon dots, and considered as the component that initially leaded us to think that the crude sample contained carbon dots based on the diffractogram. The molecular fluorophores were isolated by column chromatography and, from information obtained by characterization, we suggested that they are small hydrophobic derivatives of nitropyrene. Indeed, the FTIR and Raman characterization show the presence of nitro group, aromatic double bond carbon and in-plane carbon hydrogen bond in the different fractions. However, the less intense peaks could not be attributed. The slight difference in polarizability and optical properties could stem from these subtle chemical differences. Even though fractions F_1_ to F_4_ exhibited close hydrophobic behaviour, their distinct polarizability allowed for their separation by chromatography. The additional step in the chromatography step permitted a more advanced separation based on the small difference in polarizability of the fractions compared to the studies of Kothalawala and Batra. They both used dichloromethane and methanol as eluent for the column chromatography step whereas we used cyclohexane, dichloromethane and methanol. Indeed, the commonly used eluent system to separate hydrophobic components for silica gel chromatography is constituted of dichloromethane as the lower polarity eluent with methanol, the higher polarity eluent. But, by adding a gradient of elution using cyclohexane, an even lower polarity solvent than dichloromethane, with dichloromethane allowed us to distinguish the first four fractions. Whereas, without this modification, the first four fractions would have eluted together and we would have ended up considering them as only one fraction. Methanol was used with the intent to collect what didn't seem to migrate with dichloromethane alone. Considering the fact that the polarity strength of methanol is high, the first four fractions could have eluted as one, had we not used cyclohexane. The duration of the treatment was varied but no significant changes in the nature and properties of the products were observed in the range of duration studied. They display similar product yield and quantum yield. The first four fractions are distinguishable from the point of view of XRD and also SEM to a lesser extent. However, they display little difference from the point of view of spectroscopic techniques such as FTIR, Raman spectroscopy, UV-vis absorption. Under SEM, some fractions exhibit regular morphologies already encountered with self-assembly of pyrene derivatives.^30–34^ Powder XRD measurements suggests that samples organize in a quite ordered crystalline form. However, parameters such as the drying method, the type of solvents and the use of sonication are known to affect the formation of such supramolecular assemblies. Considering this, we cannot affirm that the samples that did not exhibit a clear morphology did so because the drying method was not adapted or they do not have a well-ordered morphology in powder form at all. F_5_, with a proportion corresponding to approximatively 10% of the sample, notably differs from the other fractions in particular by its significantly less pronounced hydrophobicity. Indeed, its elution during chromatography separation was only possible by drastically increasing the proportion of MeOH, hence the polarity of the eluent. The other fractions give clear solution in low polarity solvent even whereas solution obtained with fraction F_5_ displays cloudy solution to a certain extent, with suspended particles. The results appeared to hint that the fraction F_5_ contains or is composed of carbon entities such as carbon dots or carbon black and fluorophores free or attached to the nanoparticles. The results from the mass spectrometry analysis indicates that, even though the diffraction pattern is similar to what is expected from carbon dots, this sample could be composed of nitropyrene derivatives that differ from the other fraction that grant it a higher polarizability. The fact that it appears as an amorphous sample may be attributed to the drying process or an inherent inability to self-organize in an ordered form compared to the other fractions. Pyrene derivatives are of particular interest in supramolecular chemistry as building blocks for self-assembling. Indeed, their self-assembly behaviour in structures such as nanorods and there notable optical and electronic properties make them interesting candidates for research in this field.^36^ Through π–π stacking, nanostructures such as nanorods and nanowires were produced, because this type of interaction involves relatively high energy (*ca.* 10–70 kJ mol^−1^) and is highly directional. Templates are however needed in order to improve the crystallinity.^37,38^ Hence, studies are focusing on preparing building blocks that exhibit well-ordered self-assembly. Some studies also obtained organic nanoparticles identified as molecular fluorophores employing other precursor material than trinitropyrene like citric acid.^39–41^ This contributed to warning future studies that carefulness is necessary when evaluating samples obtained by a method commonly used to synthesize carbon dots. Though serendipitously, this study brings some information on a synthesis of luminescent self-assembling structures that displays crystallinity to a certain extent. Moreover, their hydrophobicity endows the fractions F_1_ to F_4_ great solubility in low polarity solvents. This feature and their luminescent properties could be used in ordered to prepare luminescent materials blending them in polymers such as polystyrene.

## Conclusions

Altogether, this study shows that molecules with significant fluorescent properties are produced during the solvothermal treatment of trinitropyrene. These fluorophores are highly emissive and responsible for most of the luminescence observed. Several fractions of molecular fluorophores were isolated through advanced purification steps and characterized in an attempt to identify their structures. The fractions isolated displayed similar photoluminescent properties and chemical composition but significant dissimilarities were observed from a structural point of view. Some fractions exhibited crystalline diffraction pattern, defined morphologies whereas some other samples do not show any particularly defined morphology. Molecular fluorophores were associated with derivatives of trinitropyrene, the precursor material and are thought to be mainly responsible for the luminescence fluorescence observed. This works aims to contribute to the understanding of the formation of fluorophores from a popular carbon dots precursor, trinitropyrene, used in solvothermal conditions.

## Data availability

Data supporting this article, including HRMS spectra, UV-vis absorbance spectra, Fluorescence emission spectra, FTIR spectra, Raman spectra, XRD diffraction patterns, SEM micrographs, have been included as part of the ESI.†

## Author contributions

Rayan Roch: data curation, investigation, methodology, writing – original draft, conceptualization, formal analysis. Xavier Deschanels: supervision, validation, writing – review & editing. Chandra Mohan Singaravelu: conceptualization, supervision, formal analysis. Noé André: formal analysis. Cyrielle Rey: formal analysis, supervision. Jérémy Causse: supervision, conceptualization, investigation, methodology, project administration, resources, validation, writing – review & editing.

## Conflicts of interest

There are no conflicts to declare.

## Supplementary Material

RA-014-D4RA07553F-s001
