# Supplementary material for: Evidence of the contribution of molecular fluorophores to the luminescence of carbon entities formed by solvothermal treatment of trinitropyrene
Source: RSC Adv. 2024 Dec 19;14(54):39858–66. doi: 10.1039/d4ra07553f (PMC11656156; doi:10.1039/d4ra07553f)
Supplement: RA-014-D4RA07553F-s001 [file RA-014-D4RA07553F-s001.pdf]

Supplementary Information (SI) for RSC Advances.
This journal is © The Royal Society of Chemistry 2024

Supplementary information

Evidence of the contribution of molecular fluorophores to the luminescence of carbon entities formed by solvothermal treatment of trinitropyrene

Rayan Roch*[a], Xavier Deschanels*[a], Chandra Mohan Singaravelu[a], Noé André[a], Cyrielle Rey[a] and Jérémy Causse*[a]

[a] ICSM, University of Montpellier, CEA, CNRS, ENSCM, 30207 Marcoule, France

* Corresponding authors:

Email address: rayan.roch@gmail.com (R. Roch), xavier.deschanels@cea.fr (X. Deschanels), jeremy.causse@cea.fr (J. Causse)

Figure S.I.A/ Chromatogram of the sample obtained after solvothermal treatment of TNP. The tables on the right contains information on the steps of the chromatography procedure

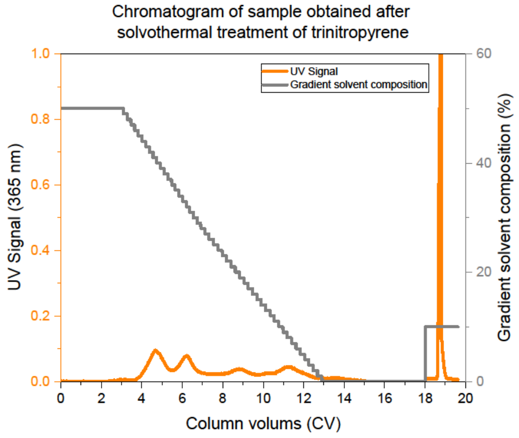

	CV	Solvents	% 2nd solvent
1	0	AB	50
2	3	AB	50
3	10	AB	0
4	5	AB	0
5	0	AC	10
6	5	AC	10

Solvent A : Dichloromethane
Solvent B : Cyclohexane
Solvent C : Methanol

S.I.B/ High Resolution Mass Spectrometry

The peak at m/z value of 214 must not be considered, it is an artifact. The solvent used for this analysis is dichloromethane. It is to be observed that the samples were not easily ionized.

Left column: positive mode; right column: negative mode

Table S.I.B/1 :

Mass of the pseudomolecular ion : 414			
	Adduct ion	Nominal adduct mass	Adduct mass
	[M+H]+	M+1	415
	[M+NH4]+	M+18	432
	[M+Na]+	M+23	437
	[2M+NH4]+	2M+NH4	846

Mass of the pseudomolecular ion : 419	Adduct ion	Nominal adduct mass	Adduct mass
	[M+H]+	M+1	420
	[M+NH4]+	M+18	437
	[M+Na]+	M+23	442
	[2M+NH4]+	2M+NH4	856

Figure S.I.B/2 ESI-HRMS Spectra of the fractions in positive and negative mode

20 min :

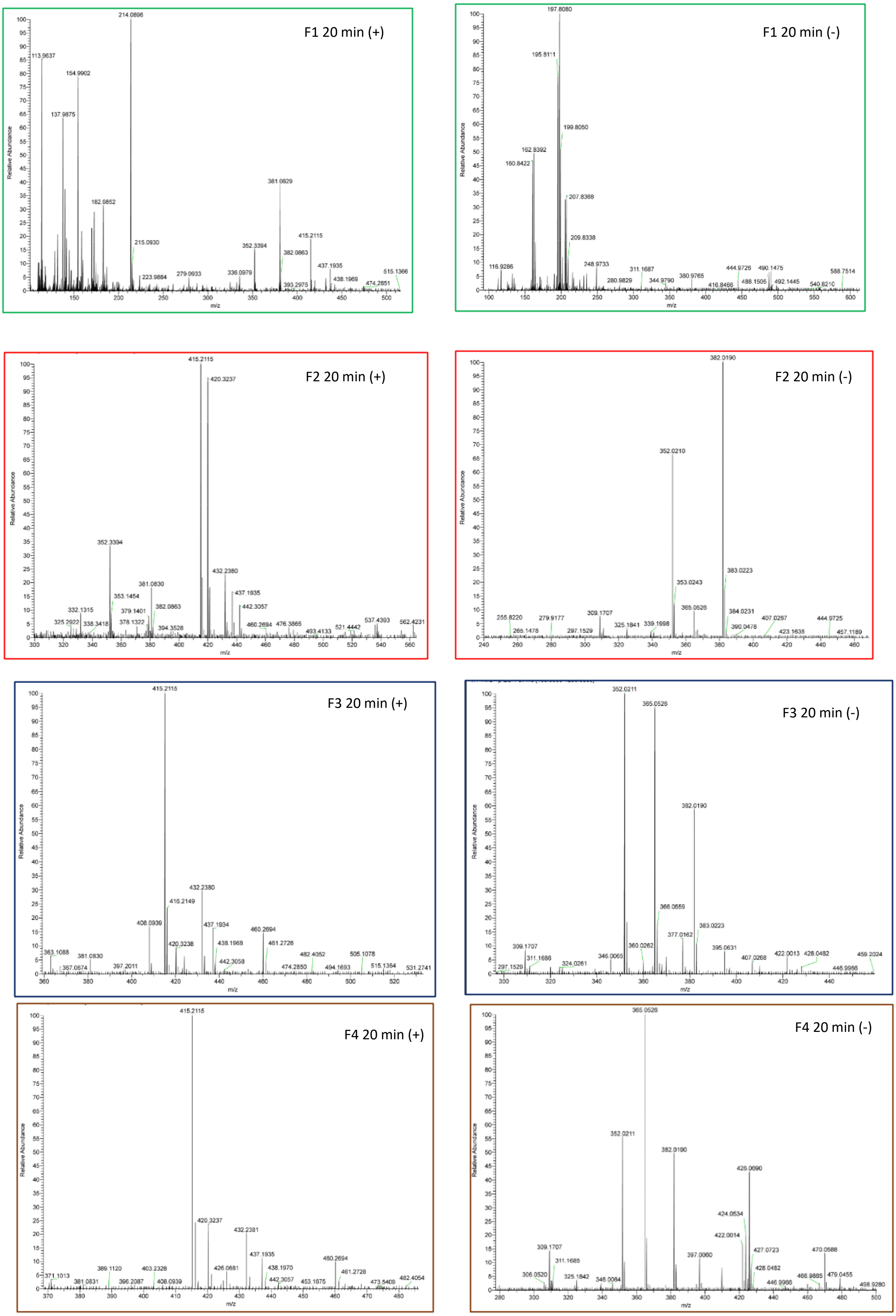

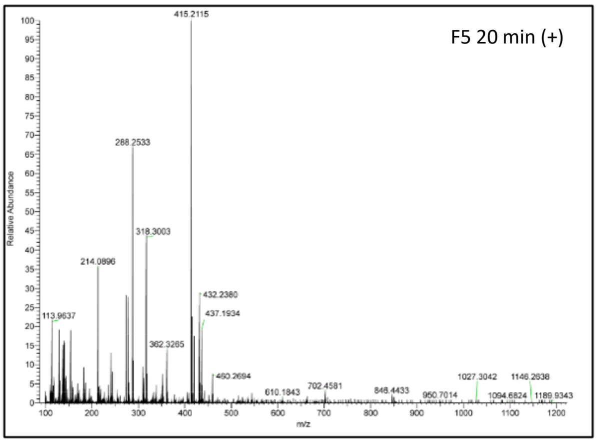
F5 20 min (+)
Relative Abundance
m/z
415.2115
288.2533
318.3003
214.0896
113.9637
362.3265
432.2380
437.1934
460.2694
610.1843
702.4581
846.4433
950.7014
1027.3042
1146.2638
1094.6824
1189.9343

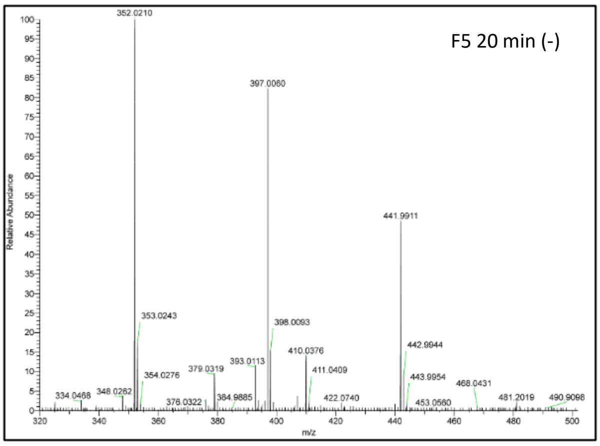
F5 20 min (-)
Relative Abundance
m/z
352.0210
397.0060
441.9911
353.0243
398.0093
410.0376
393.0113
379.0319
411.0409
354.0276
334.0468
348.0282
378.0322
384.9885
422.0740
442.9944
443.9954
468.0431
453.0560
481.2019
490.9098

40 min :

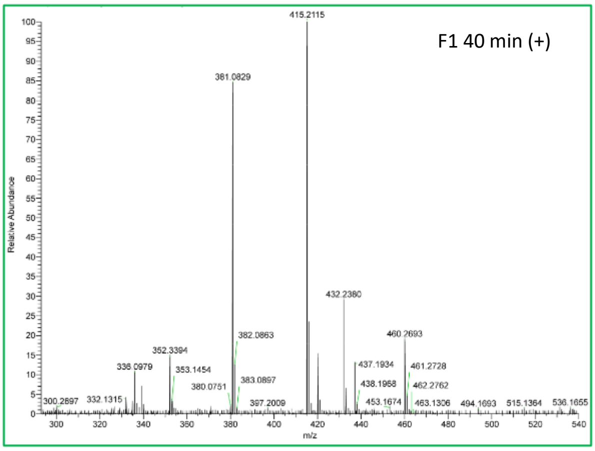
F1 40 min (+)
Relative Abundance
m/z
415.2115
381.0829
432.2380
460.2693
382.0863
352.3394
336.0979
353.1454
380.0751
383.0897
437.1934
461.2728
438.1968
462.2762
300.2897
332.1315
397.2009
453.1674
463.1306
494.1693
515.1364
536.1655

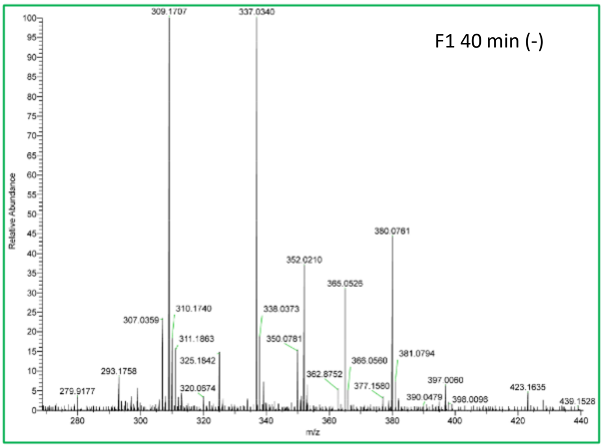
F1 40 min (-)
Relative Abundance
m/z
309.1707
337.0340
380.0761
352.0210
365.0525
310.1740
338.0373
307.0359
311.1863
350.0781
325.1842
366.0560
381.0794
293.1758
362.8752
377.1580
397.0060
279.9177
320.0574
390.0479
398.0098
423.1635
439.1528

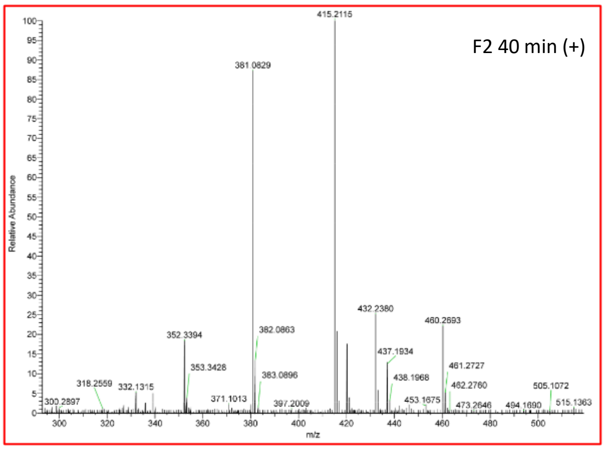
F2 40 min (+)
Relative Abundance
m/z
415.2115
381.0829
432.2380
460.2693
352.3394
382.0863
437.1934
353.3428
383.0896
438.1968
461.2727
462.2760
318.2559
332.1315
505.1072
300.2897
371.1013
397.2009
453.1675
473.2646
494.1690
515.1363

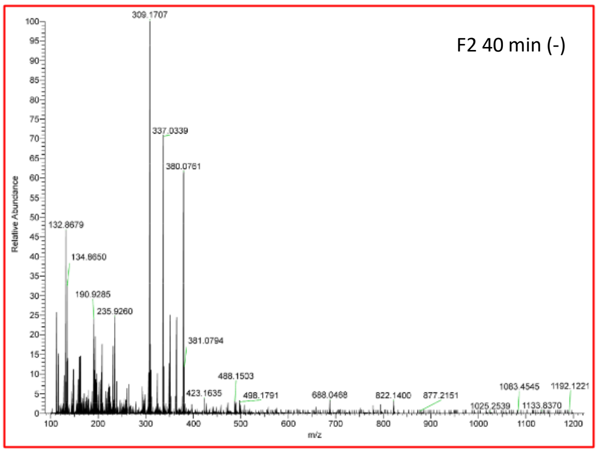
F2 40 min (-)
Relative Abundance
m/z
309.1707
337.0339
380.0761
132.8679
134.8650
190.9285
235.9260
381.0794
488.1503
423.1635
498.1791
688.0468
822.1400
877.2151
1083.4545
1192.1221
1025.2539
1133.8370

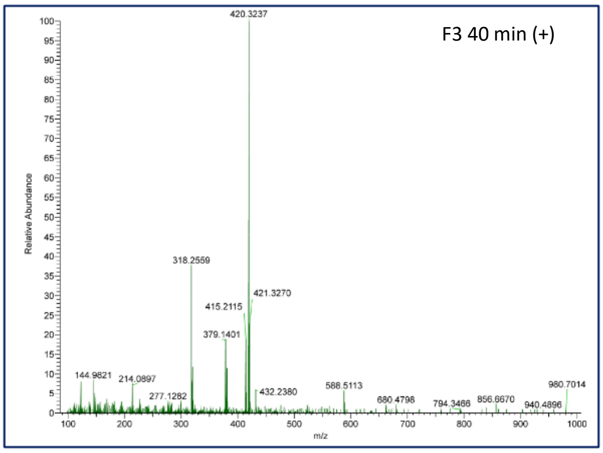
F3 40 min (+)
Relative Abundance
m/z
420.3237
318.2559
421.3270
415.2115
379.1401
144.9821
214.0897
277.1282
432.2380
588.5113
680.4798
794.3466
856.6670
940.4896
980.7014

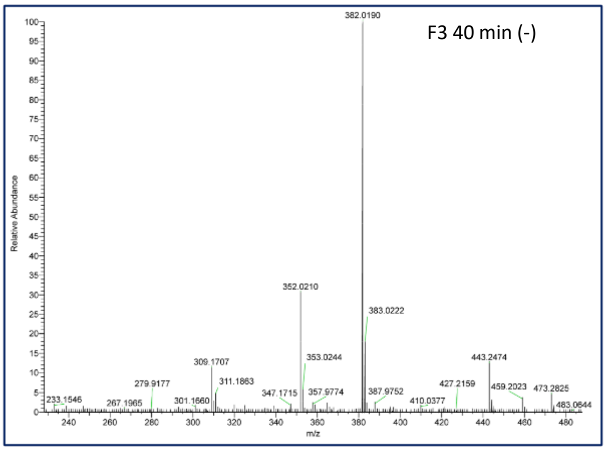
F3 40 min (-)
Relative Abundance
m/z
382.0190
352.0210
383.0222
353.0244
443.2474
309.1707
311.1863
279.9177
427.2159
459.2023
473.2825
233.1546
267.1965
301.1660
347.1715
357.9774
387.9752
410.0377
483.0644

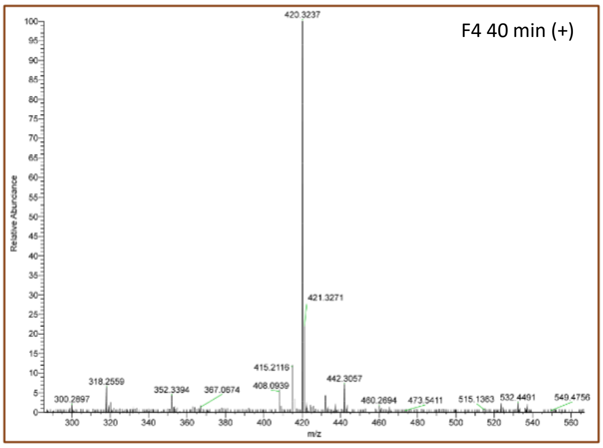
F4 40 min (+)
420.3237
421.3271
415.2116
408.0939
442.3057
318.2559
352.3394
367.0674
300.2897
460.2694
473.5411
515.1363
532.4491
549.4756
Relative Abundance
m/z

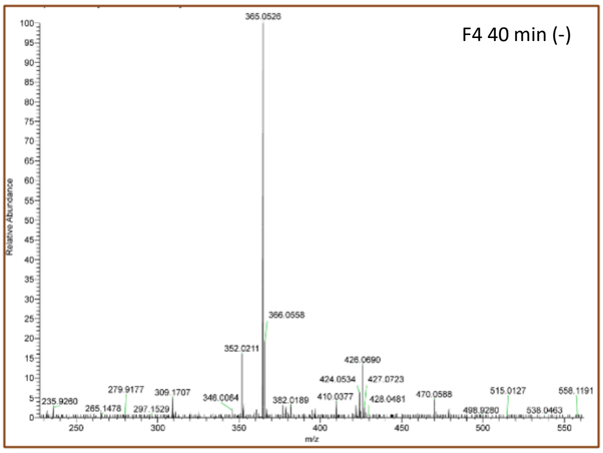
F4 40 min (-)
365.0526
366.0558
352.0211
426.0690
424.0534
427.0723
279.9177
309.1707
346.0064
382.0189
410.0377
428.0481
470.0588
515.0127
558.1191
235.9260
265.1478
297.1529
498.9280
538.0463
Relative Abundance
m/z

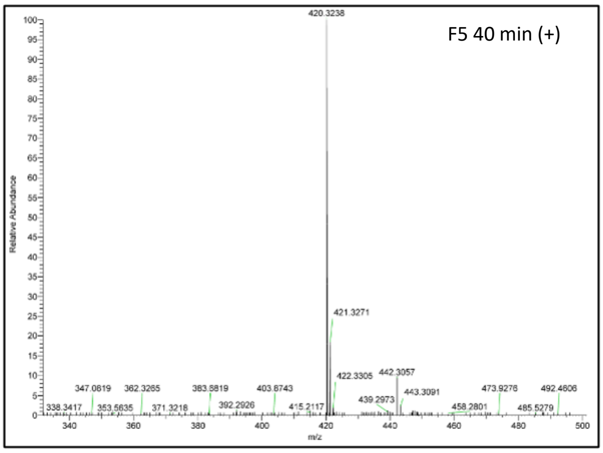
F5 40 min (+)
420.3238
421.3271
422.3305
442.3057
443.3091
439.2973
347.0819
362.3265
383.5819
403.8743
473.9276
492.4606
338.3417
353.5635
371.3218
392.2926
415.2117
458.2801
485.5279
Relative Abundance
m/z

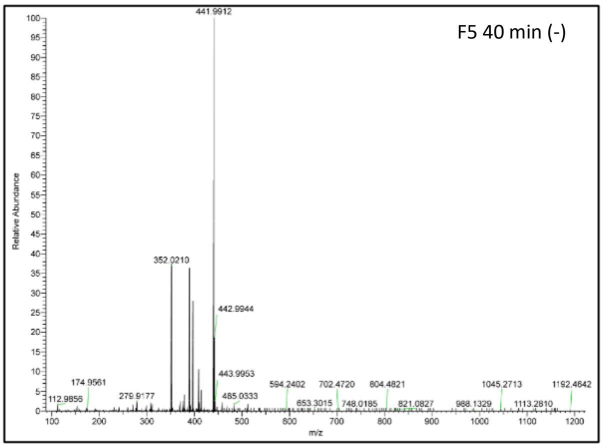
F5 40 min (-)
441.9912
352.0210
442.9944
443.9953
485.0333
174.9561
112.9856
279.9177
594.2402
702.4720
804.4821
1045.2713
1192.4642
653.3015
748.0185
821.0827
988.1329
1113.2810
Relative Abundance
m/z

60 min :

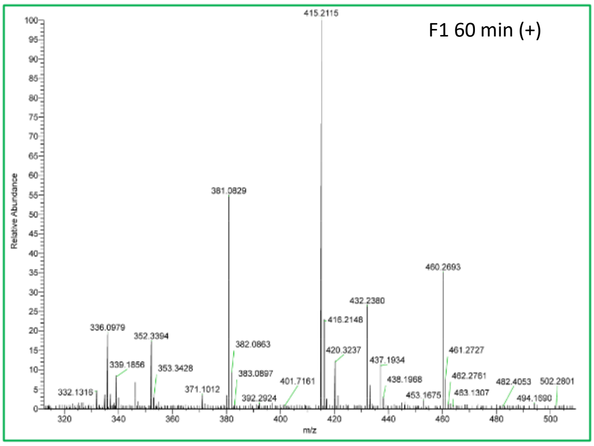
F1 60 min (+)
415.2115
381.0829
460.2693
432.2380
416.2148
336.0979
352.3394
382.0863
420.3237
437.1934
461.2727
339.1856
353.3428
383.0897
438.1968
462.2761
401.7161
482.4053
502.2801
332.1316
371.1012
392.2924
453.1675
463.1307
494.1890
Relative Abundance
m/z

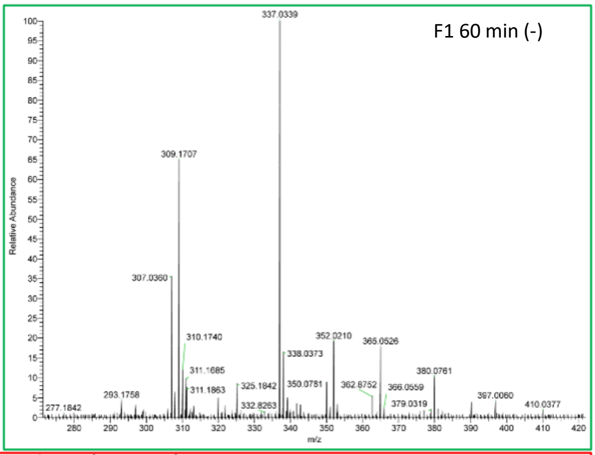
F1 60 min (-)
337.0339
309.1707
307.0360
310.1740
352.0210
365.0526
338.0373
311.1685
380.0761
325.1842
350.0781
362.8752
366.0559
311.1863
293.1758
397.0060
332.8263
379.0319
410.0377
277.1842
Relative Abundance
m/z

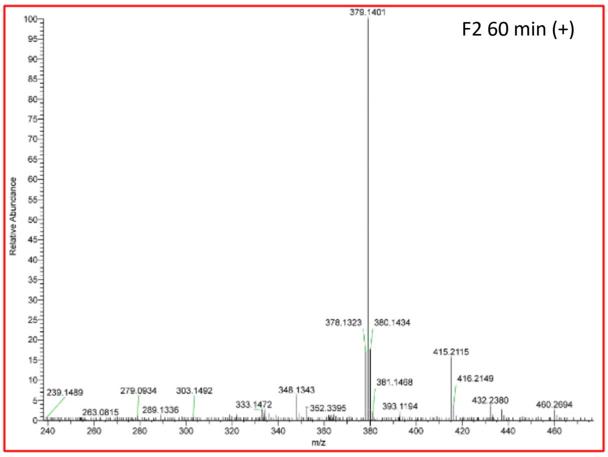
F2 60 min (+)
379.1401
378.1323
380.1434
415.2115
416.2149
381.1468
239.1489
279.0934
303.1492
348.1343
333.1472
263.0815
289.1336
352.3395
393.1194
432.2380
460.2694
Relative Abundance
m/z

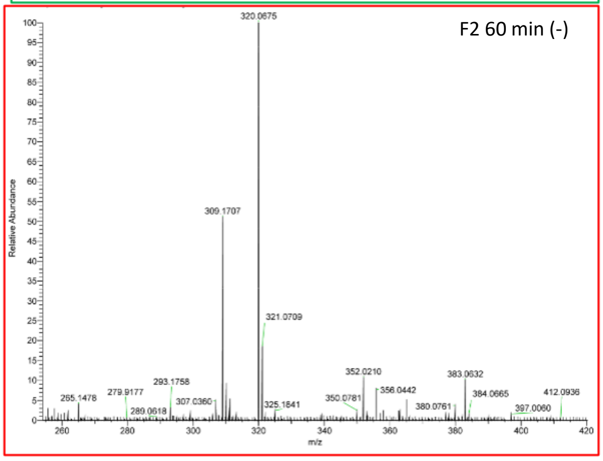
F2 60 min (-)
320.0675
309.1707
321.0709
352.0210
383.0632
356.0442
265.1478
279.9177
293.1758
384.0665
412.0936
307.0360
325.1841
350.0781
280.0618
380.0761
397.0060
Relative Abundance
m/z

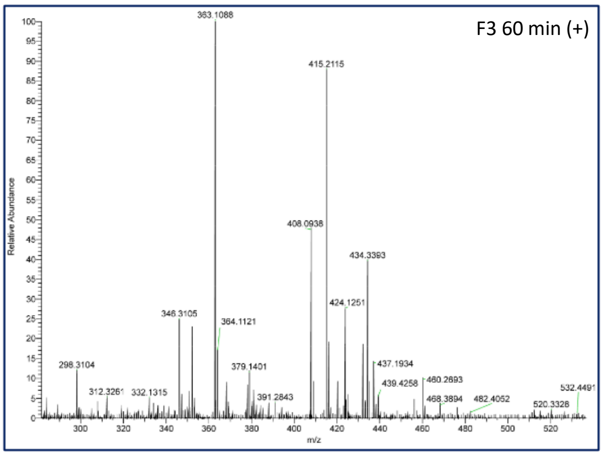
F3 60 min (+)
Relative Abundance
m/z
363.1088
415.2115
408.0938
434.3393
424.1251
346.3105
364.1121
298.3104
379.1401
437.1934
480.2893
439.4258
312.3261
332.1315
391.2843
468.3894
482.4052
532.4491
520.3328

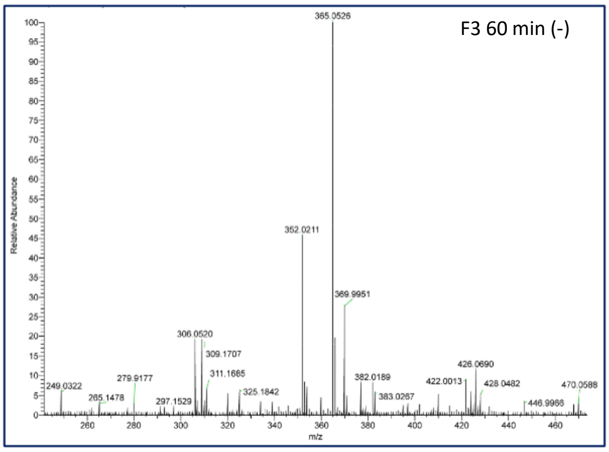
F3 60 min (-)
Relative Abundance
m/z
365.0526
352.0211
369.9951
306.0520
309.1707
311.1685
249.0322
279.9177
265.1478
297.1529
325.1842
382.0189
383.0267
426.0690
422.0013
428.0482
470.0588
446.9968

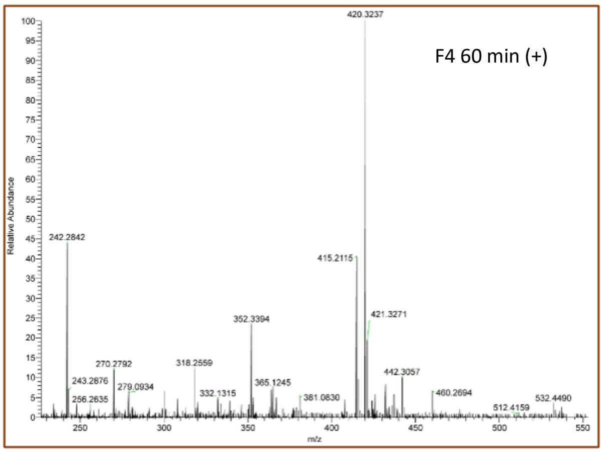
F4 60 min (+)
Relative Abundance
m/z
420.3237
242.2842
415.2115
421.3271
352.3394
270.2792
318.2559
243.2876
279.0934
256.2635
332.1315
365.1245
381.0830
442.3057
460.2694
512.4159
532.4490

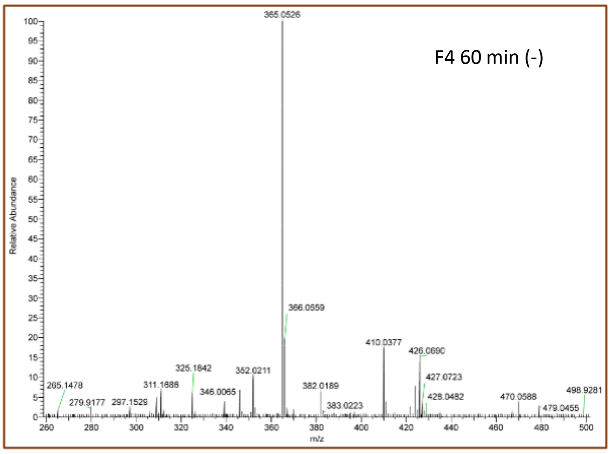
F4 60 min (-)
Relative Abundance
m/z
365.0526
366.0559
410.0377
426.0690
325.1842
352.0211
265.1478
311.1688
346.0065
382.0189
427.0723
428.0482
279.9177
297.1529
383.0223
470.0588
498.9281
479.0455

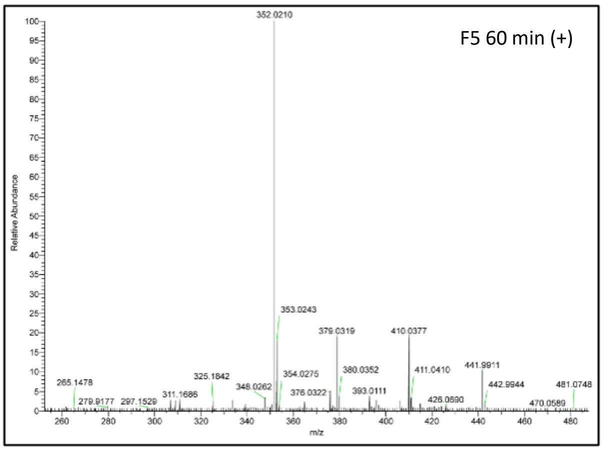
F5 60 min (+)
Relative Abundance
m/z
352.0210
353.0243
379.0319
410.0377
441.9911
325.1842
354.0275
380.0352
411.0410
265.1478
348.0262
442.9944
481.0748
376.0322
393.0111
279.9177
297.1529
311.1686
426.0690
470.0589

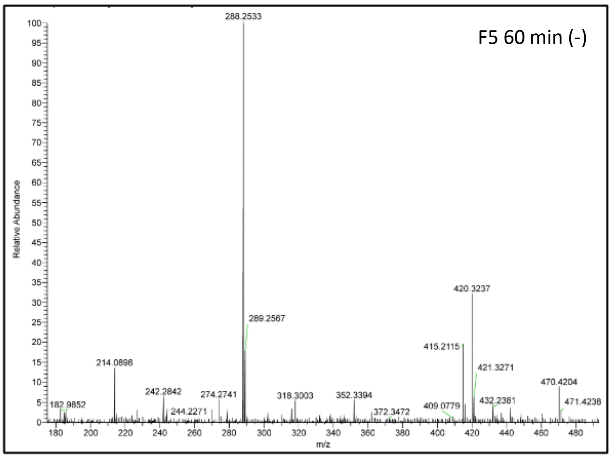
F5 60 min (-)
Relative Abundance
m/z
288.2533
420.3237
289.2567
415.2115
214.0896
421.3271
470.4204
242.2842
274.2741
318.3003
352.3394
471.4238
182.9852
244.2271
372.3472
409.0779
432.2381

Figure S.I.C/ Fourier Transform InfraRed spectroscopy

FTIR Spectra of the fractions for 20, 40 and 60 minutes stacked with TNP FTIR spectra

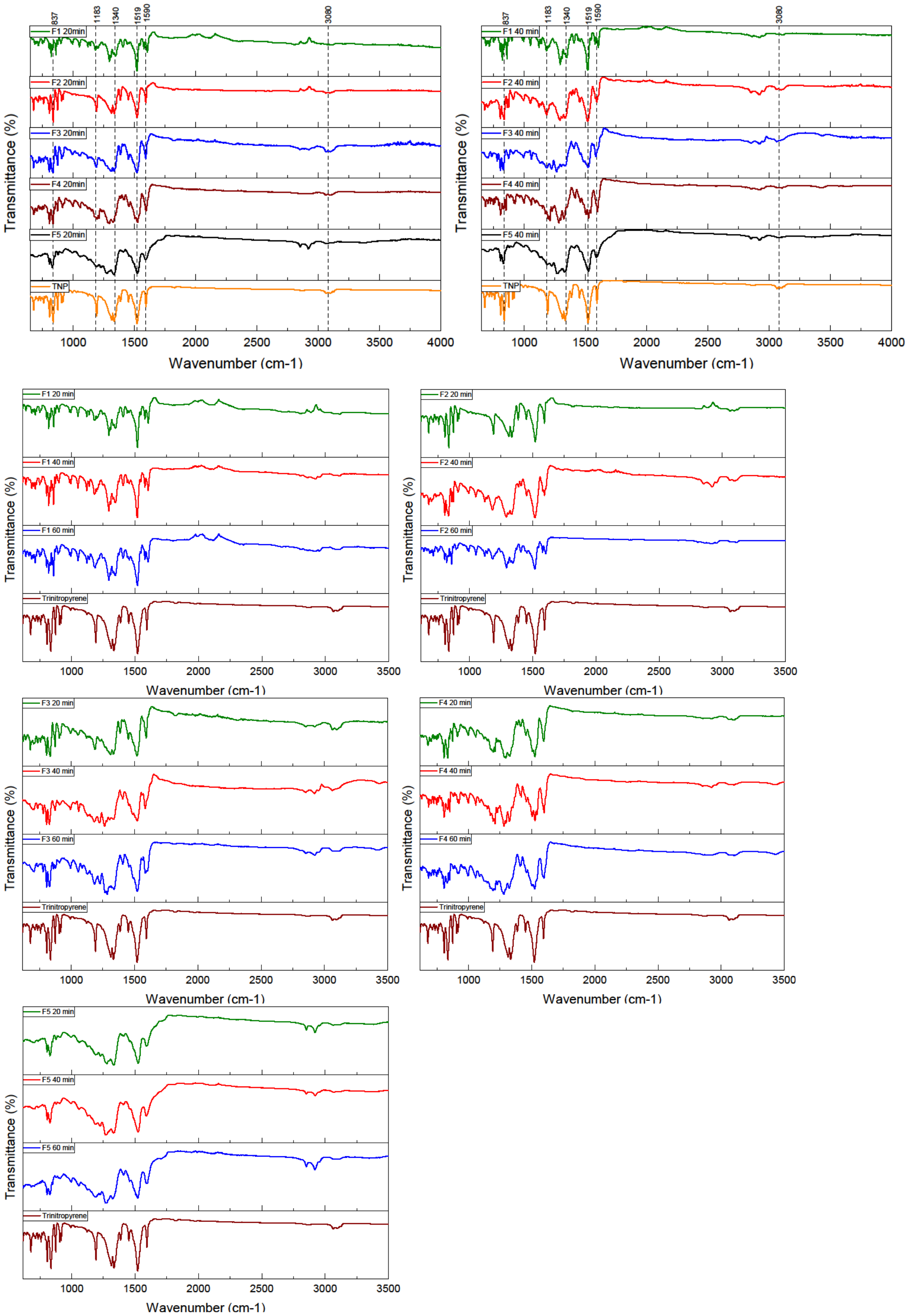

Figure S.I.D/ Raman spectroscopy

Raman spectra of TNP and the fractions for durations of 20 and 40 minutes of solvothermal treatment

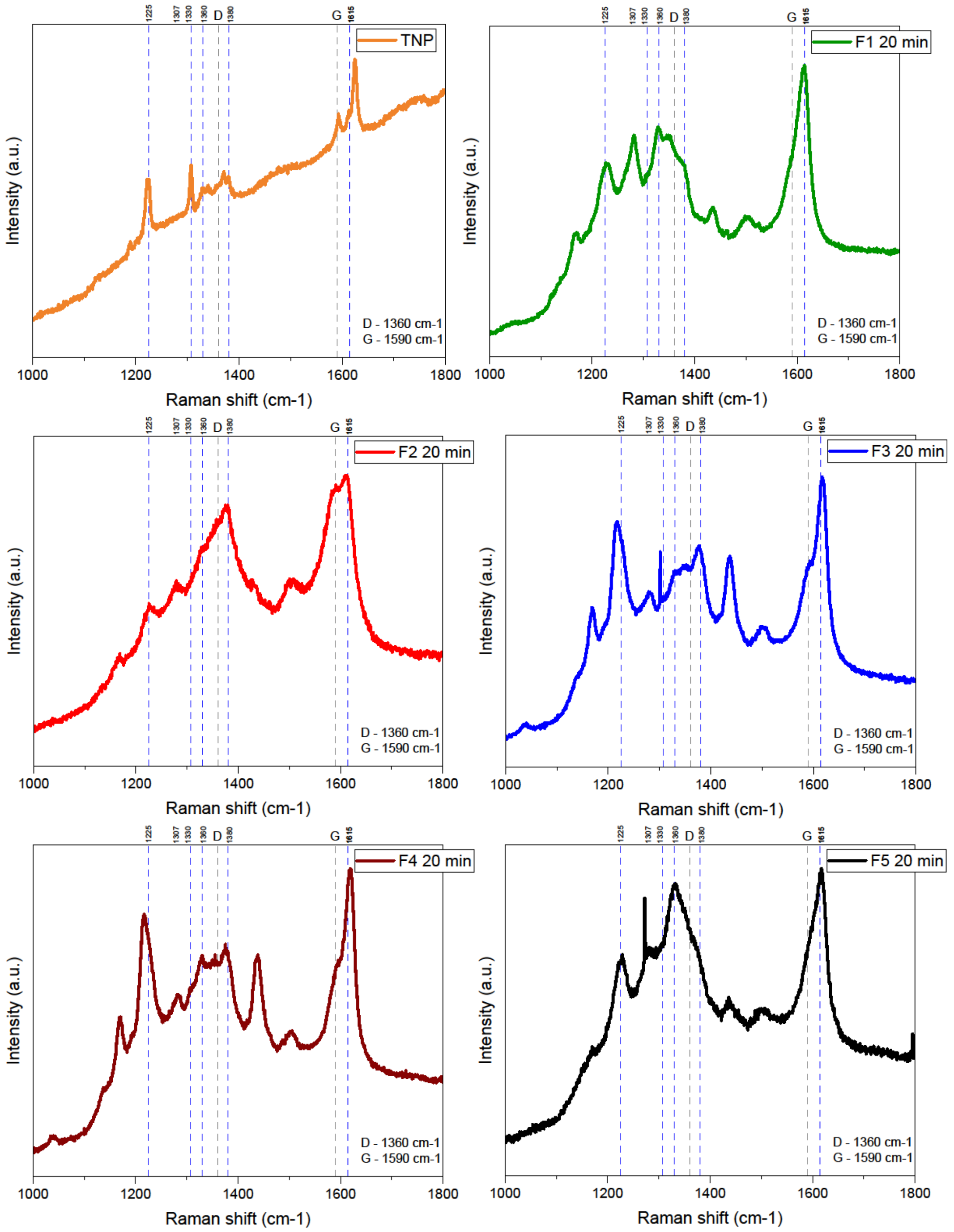

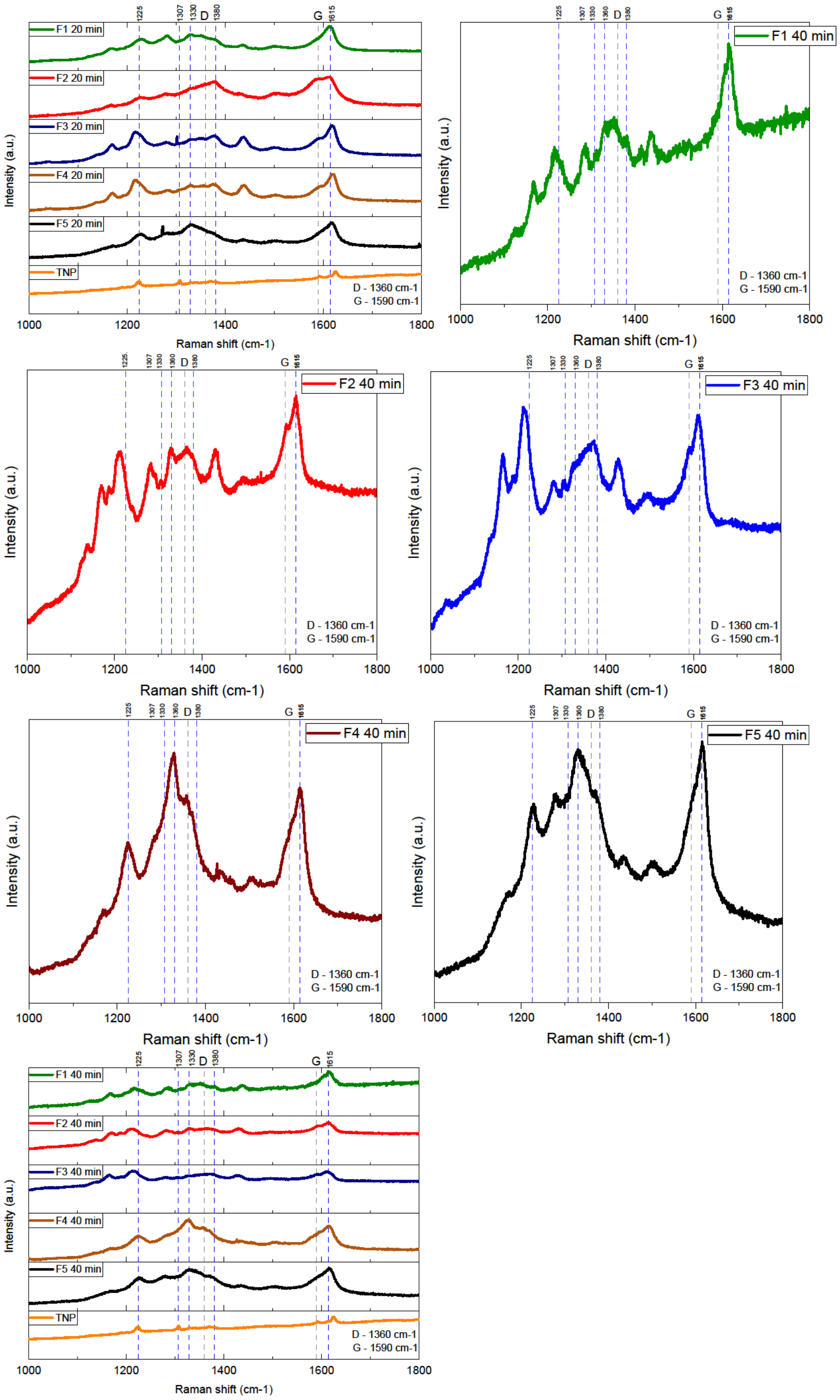
F1 20 min
F2 20 min
F3 20 min
F4 20 min
F5 20 min
TNP
1225
1307
1330
D
1380
G
1615
D - 1360 cm-1
G - 1590 cm-1
Intensity (a.u.)
Raman shift (cm-1)
1000
1200
1400
1600
1800
F1 40 min
F2 40 min
F3 40 min
F4 40 min
F5 40 min
1360

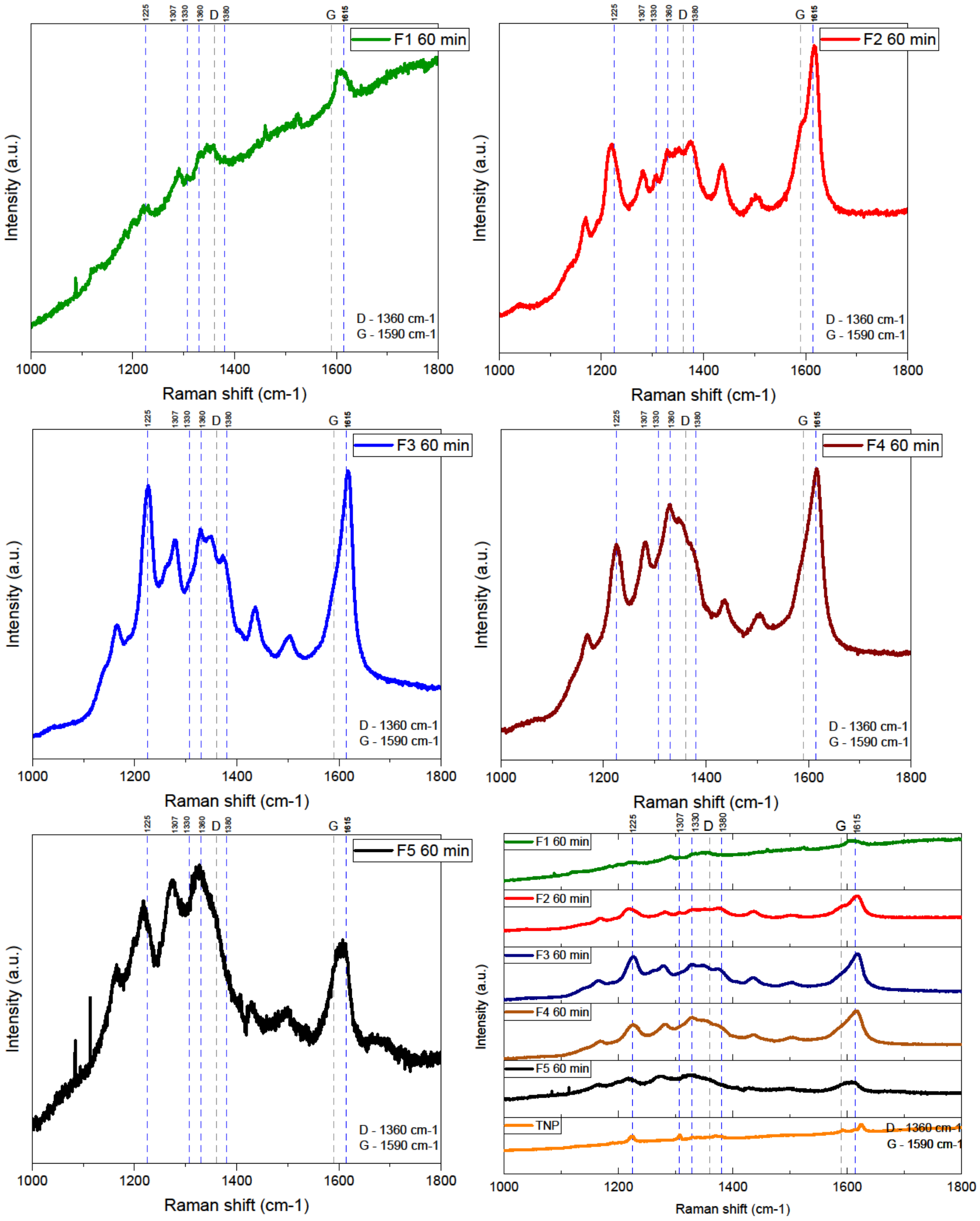
F1 60 min
F2 60 min
F3 60 min
F4 60 min
F5 60 min
TNP
Intensity (a.u.)
Raman shift (cm-1)
D - 1360 cm-1
G - 1590 cm-1
1225
1307
1330
1360
1380
1615
D
G
1000
1200
1400
1600
1800

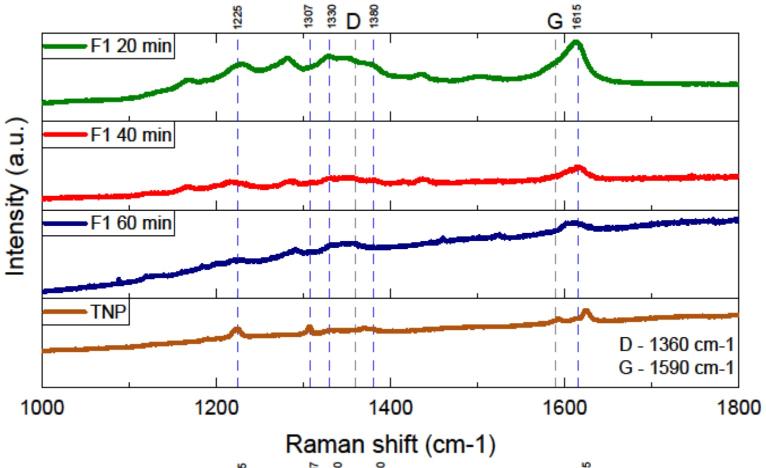
1225
1307
1330
D
1380
G
1615
F1 20 min
F1 40 min
F1 60 min
TNP
D - 1360 cm-1
G - 1590 cm-1
Intensity (a.u.)
1000
1200
1400
1600
1800
Raman shift (cm-1)

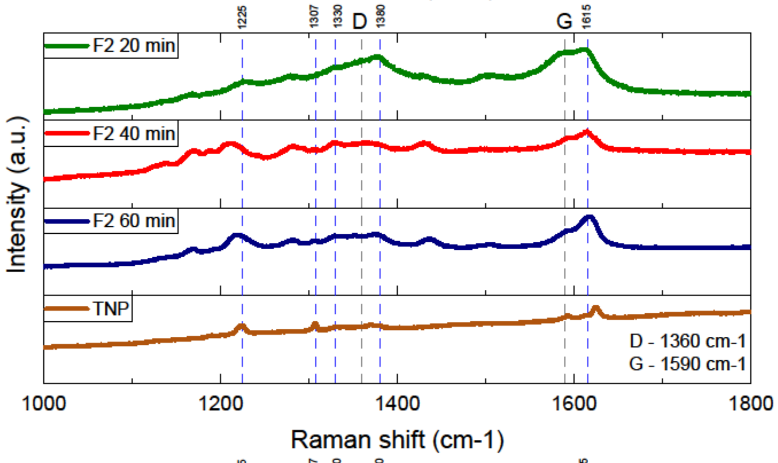
1225
1307
1330
D
1380
G
1615
F2 20 min
F2 40 min
F2 60 min
TNP
D - 1360 cm-1
G - 1590 cm-1
Intensity (a.u.)
1000
1200
1400
1600
1800
Raman shift (cm-1)

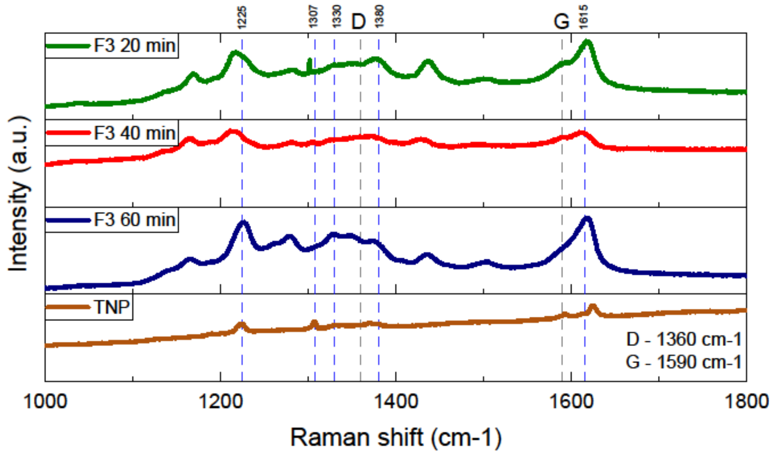
1225
1307
1330
D
1380
G
1615
F3 20 min
F3 40 min
F3 60 min
TNP
D - 1360 cm-1
G - 1590 cm-1
Intensity (a.u.)
1000
1200
1400
1600
1800
Raman shift (cm-1)

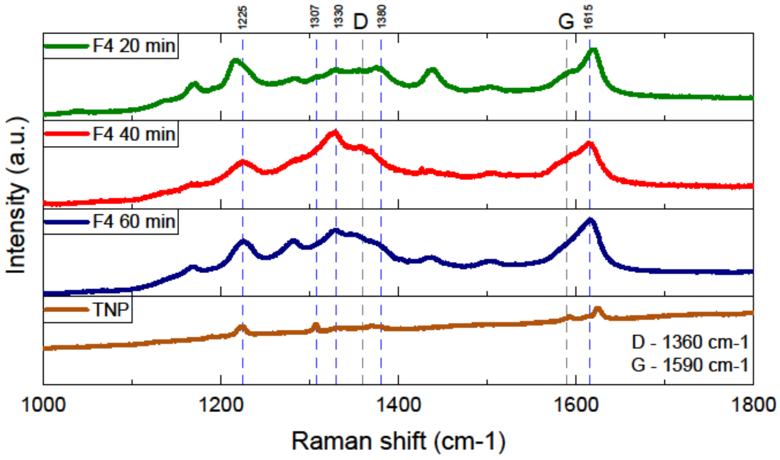
1225
1307
1330
D
1380
G
1615
F4 20 min
F4 40 min
F4 60 min
TNP
D - 1360 cm-1
G - 1590 cm-1
Intensity (a.u.)
1000
1200
1400
1600
1800
Raman shift (cm-1)

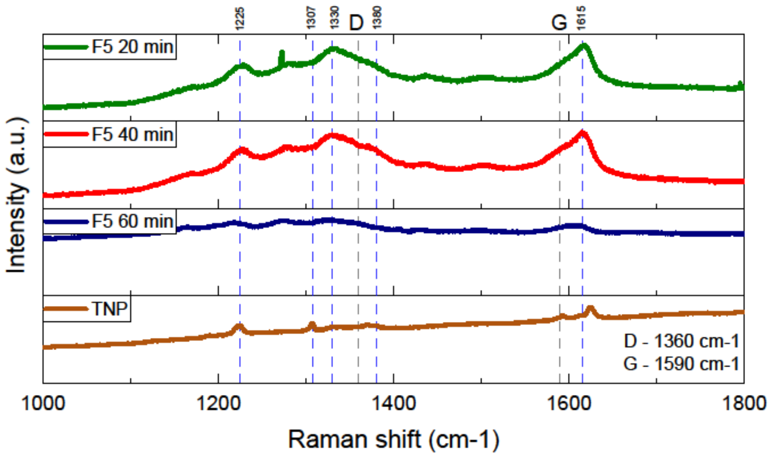

Figure S.I.E. X-ray Diffraction

Diffraction pattern (individual and stacked) of the fractions F1 to F5 with (left column) and without (right column) the background (60 minutes duration of solvothermal treatment)

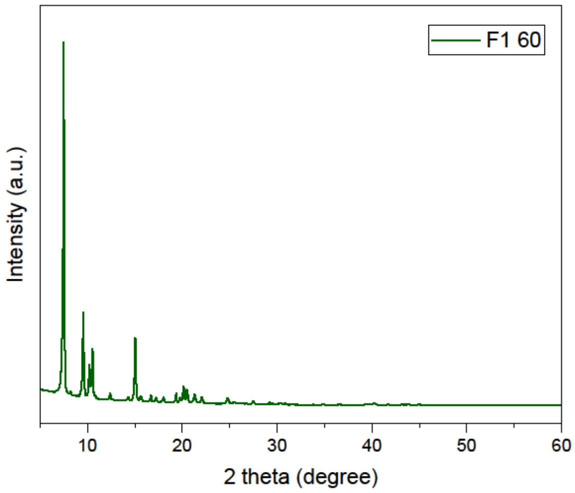

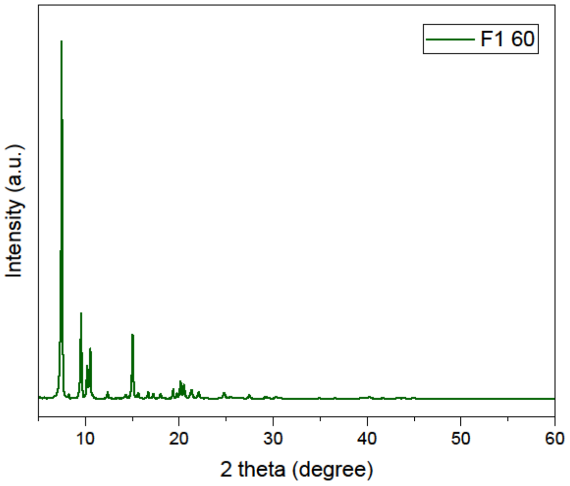

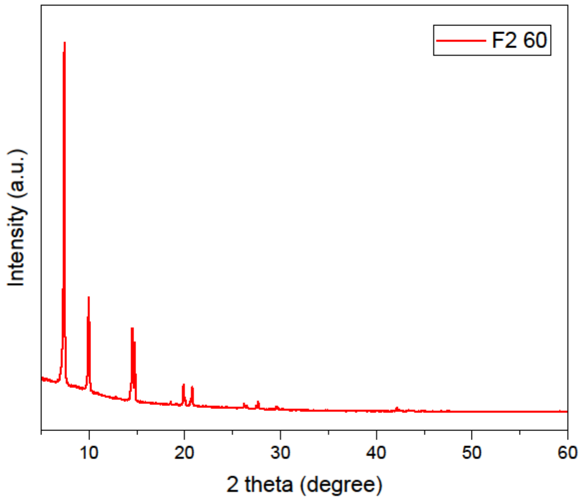

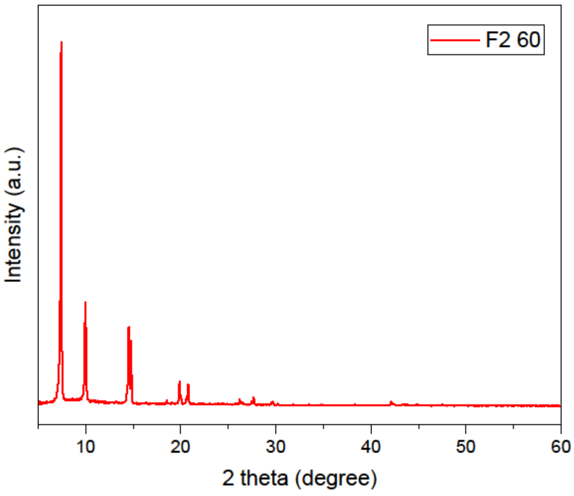

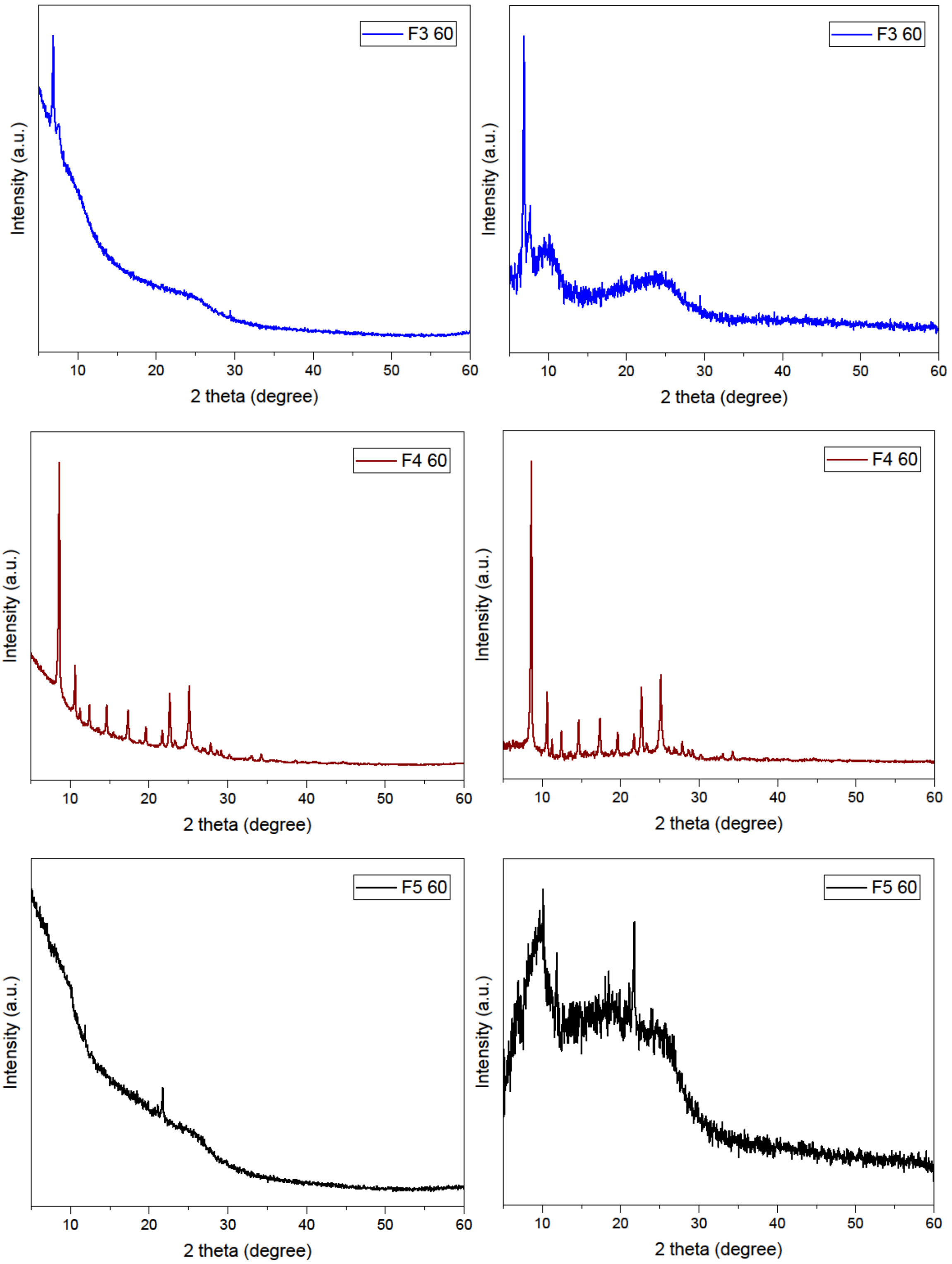
F3 60
Intensity (a.u.)
2 theta (degree)
F3 60
Intensity (a.u.)
2 theta (degree)
F4 60
Intensity (a.u.)
2 theta (degree)
F4 60
Intensity (a.u.)
2 theta (degree)
F5 60
Intensity (a.u.)
2 theta (degree)
F5 60
Intensity (a.u.)
2 theta (degree)

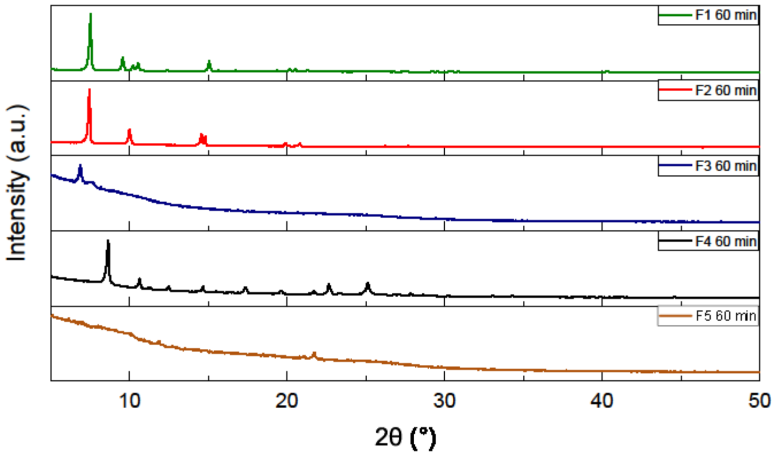
F1 60 min
F2 60 min
F3 60 min
F4 60 min
F5 60 min
Intensity (a.u.)
10
20
30
40
50
2θ (°)

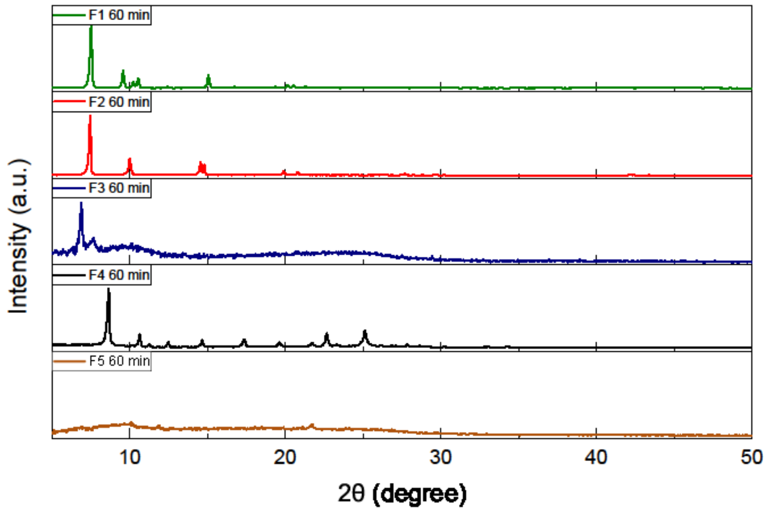
F1 60 min
F2 60 min
F3 60 min
F4 60 min
F5 60 min
Intensity (a.u.)
10
20
30
40
50
2θ (degree)

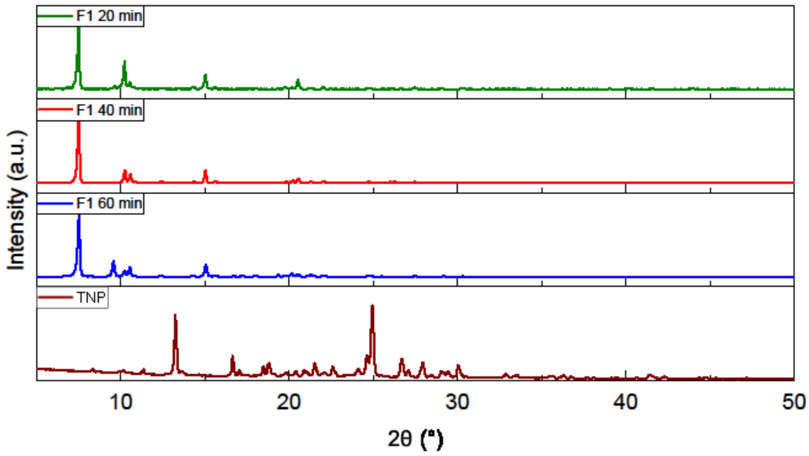
F1 20 min
F1 40 min
F1 60 min
TNP
Intensity (a.u.)
10
20
30
40
50
2θ (°)

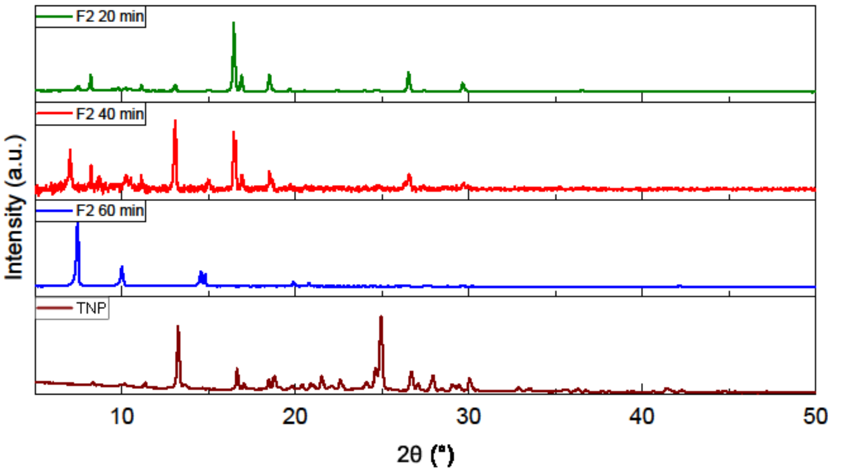
F2 20 min
F2 40 min
F2 60 min
TNP
Intensity (a.u.)
10
20
30
40
50
2θ (°)

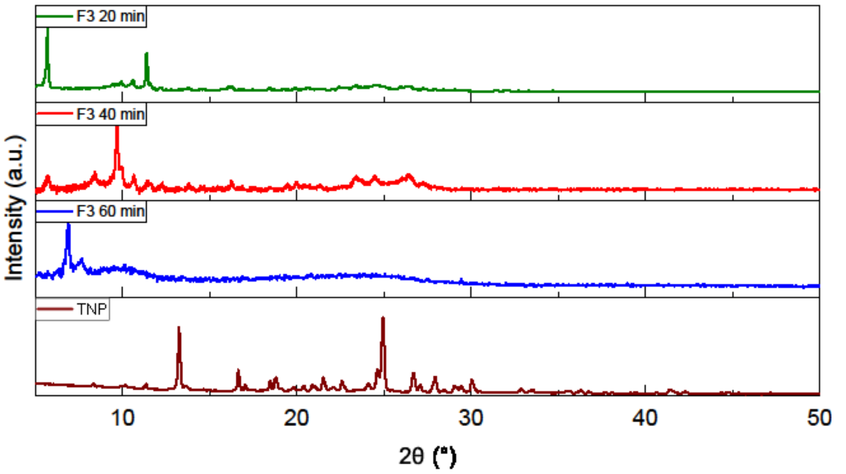
F3 20 min
F3 40 min
F3 60 min
TNP
Intensity (a.u.)
10
20
30
40
50
2θ (°)

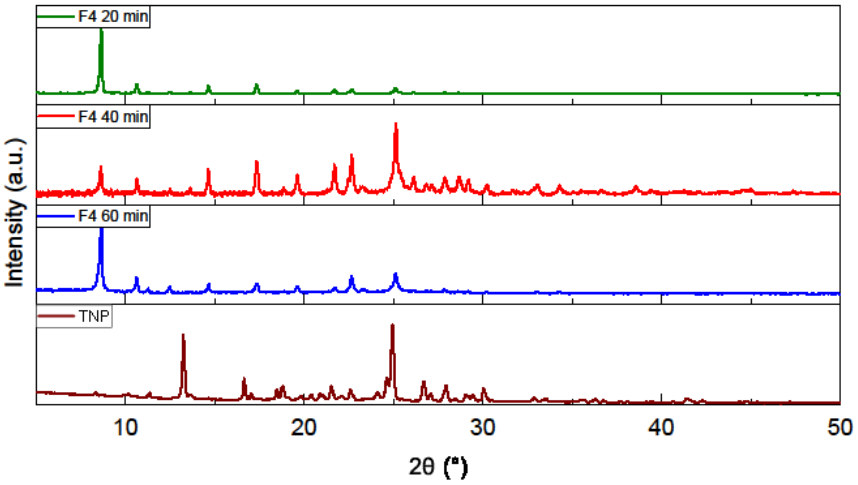
F4 20 min
F4 40 min
F4 60 min
TNP
Intensity (a.u.)
10
20
30
40
50
2θ (°)

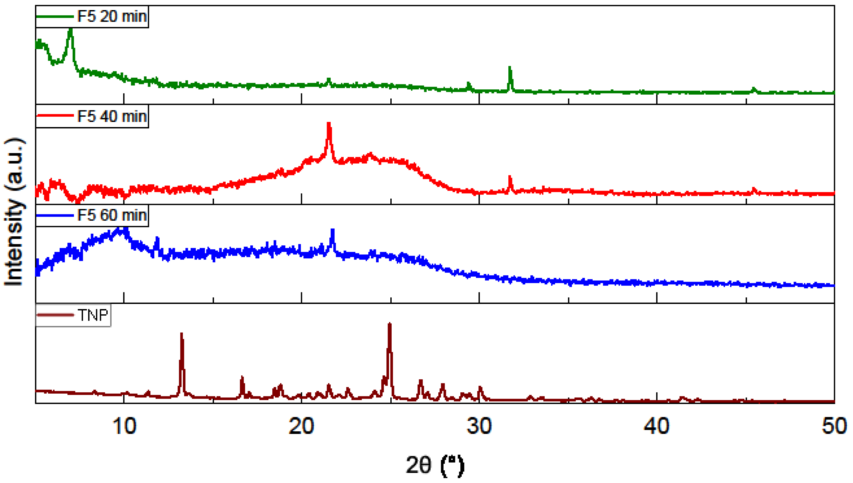
F5 20 min
F5 40 min
F5 60 min
TNP
Intensity (a.u.)
10
20
30
40
50
2θ (°)

S.I.F/ SEM

For 20 min synthesis, the fraction F_1 20 shows tangled, stacked thread-like structure of various size (µm size) and diameter. Some of the threads are bent. Fraction F_2 20 displays straight rods-like. They are regular and larger than the thread from F_1 20. If we focus on the surface of the rods, we observe smaller stacked pieces and irregularities. Fractions F_3 20 and F_4 20 exhibit small pieces of irregular size and shape. F_5 20 displays small and large pieces of irregular size and shape. Some of them look like the other four fractions. Overall, the fractions are µm size. For 40 min synthesis, the fraction F_1 40 shows tangled, stacked thread-like structure of various size (µm size) and diameter similarly to the F_1 20. Some of the threads are bent. Fraction F_2 40 displays rods and sheet-like structure. F_2 40 structure is very different from F_2 20. They are irregular and similar in size to F_1 40. If we focus on the surface of the rods, we observe smaller stacked pieces and irregularities. Fractions F_3 exhibit stacked spike-like structure of varying size. F_4 does not have a clear morphology as it contains rods, threads, and sheets-like pieces. Overall, some fractions display quite regular structure. This imply that the sample structure is organized on a long range. This is in accordance with the XRD results that suggest that these samples are quite crystalline. This is not something expected for carbon dots but these morphologies are expected for organic molecules. Moreover, reports of pyrene derivatives that self-assembled to form supramolecular object such as nanorods have been made[1,2,3,]. This suggest, like the XRD and the HRMS results, that the samples are more likely composed of a pool of molecular fluorophores.

Figure S.I.F/1 SEM images of the fractions from F1 to F5 for 20, 40 and 60 minutes of solvothermal treatment

20 min :

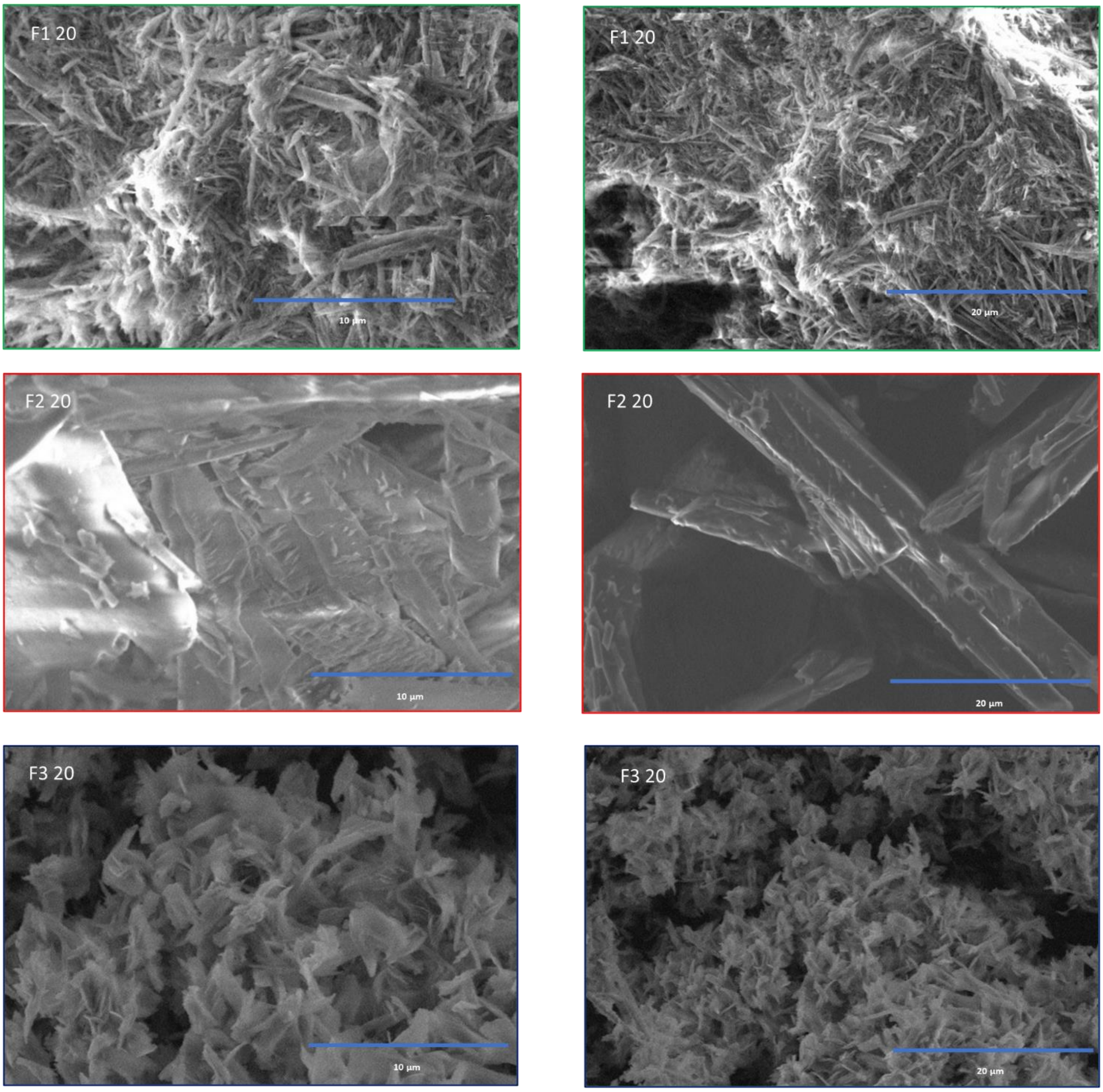

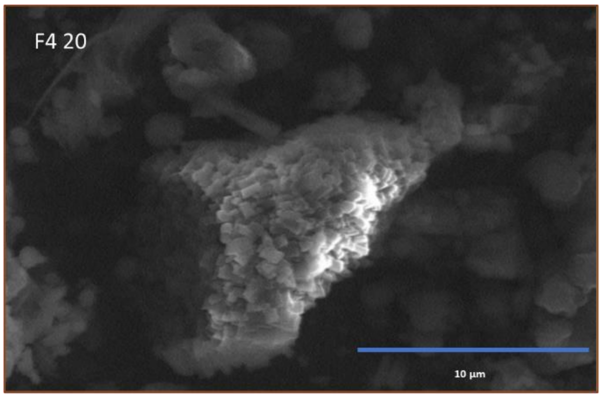
F4 20
10 µm

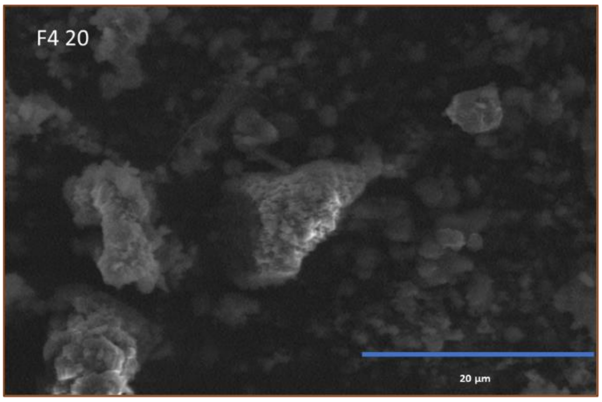
F4 20
20 µm

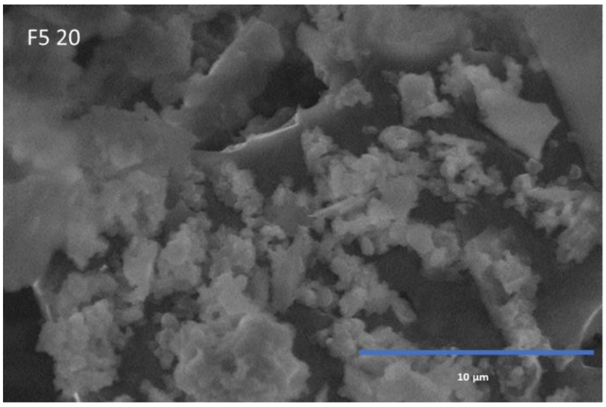
F5 20
10 µm

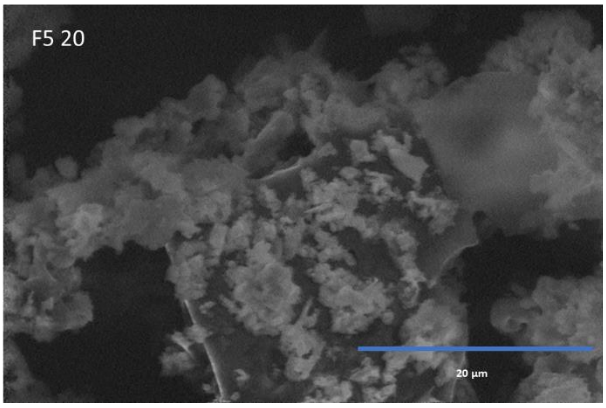
F5 20
20 µm

40 min :

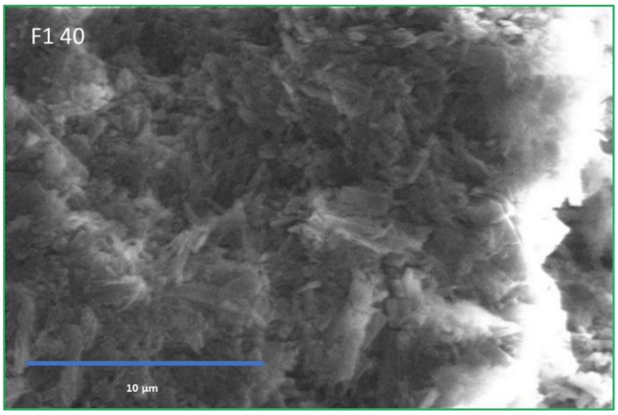
F1 40
10 µm

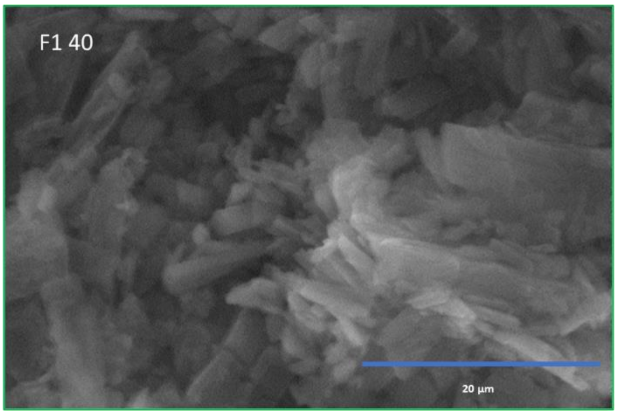
F1 40
20 µm

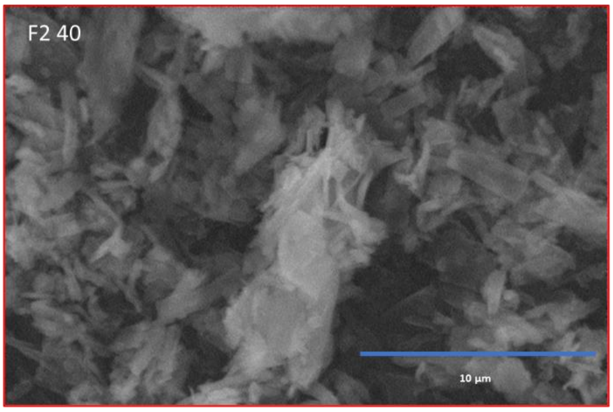
F2 40
10 µm

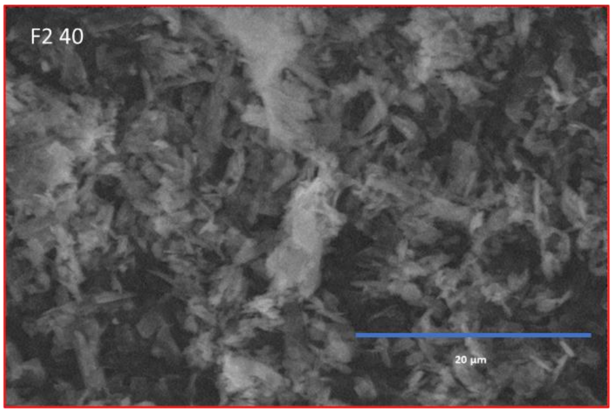
F2 40
20 µm

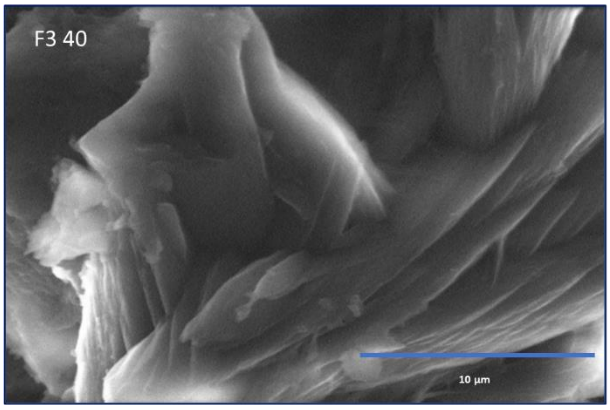
F3 40
10 µm
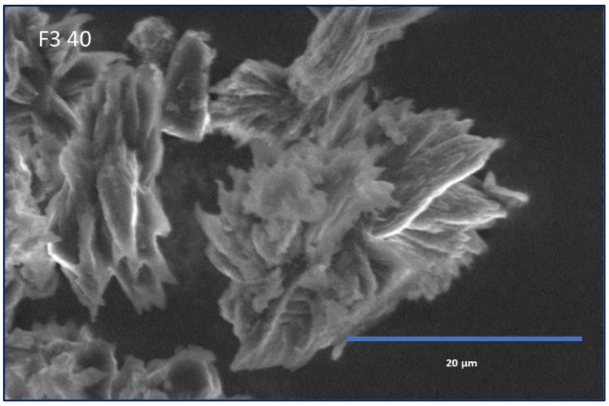
F3 40
20 µm
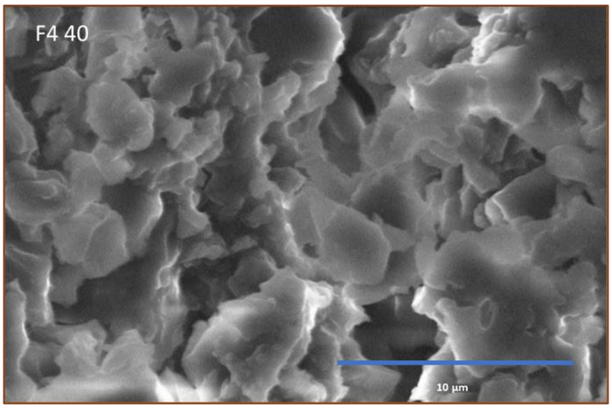
F4 40
10 µm
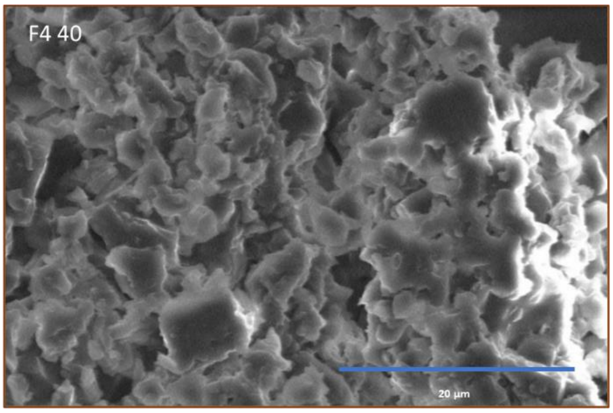
F4 40
20 µm
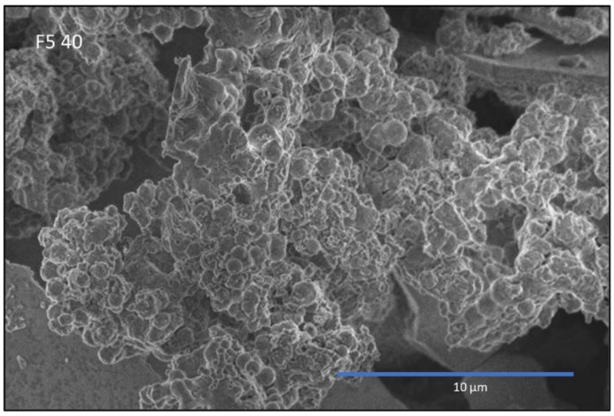
F5 40
10 µm
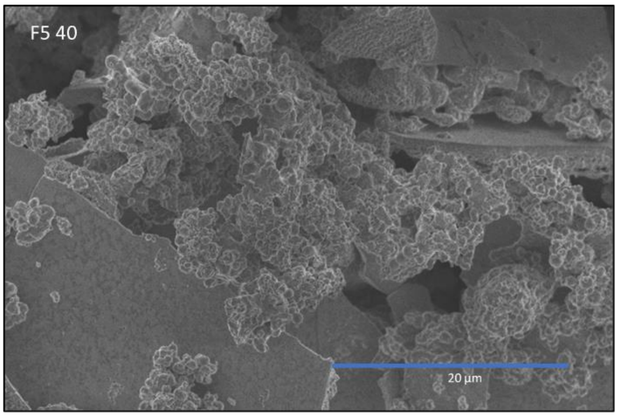
F5 40
20 µm

60 min :

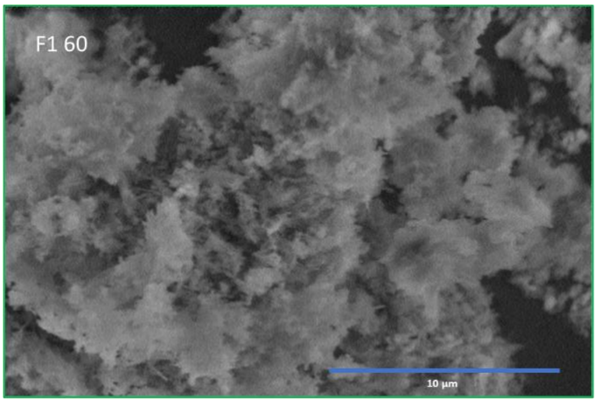
F1 60
10 µm
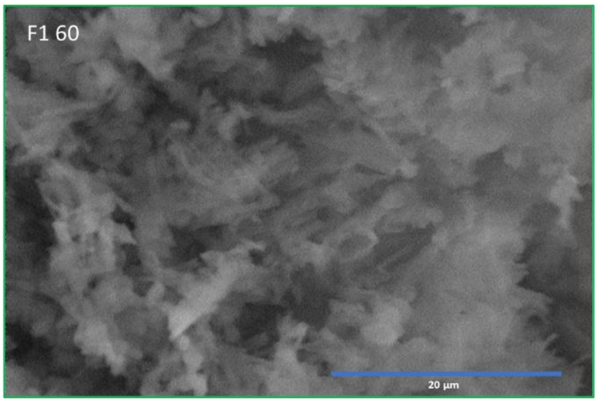
F1 60
20 µm

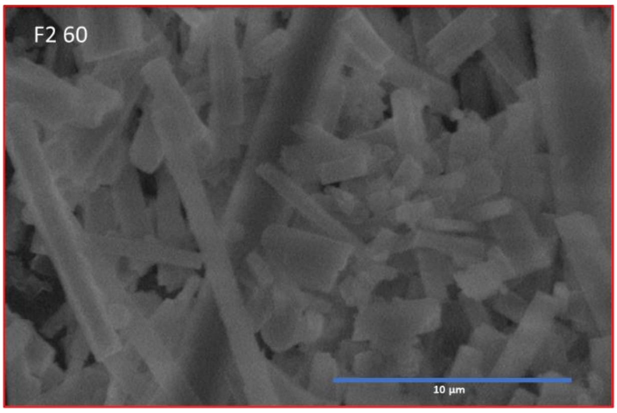
F2 60
10 µm

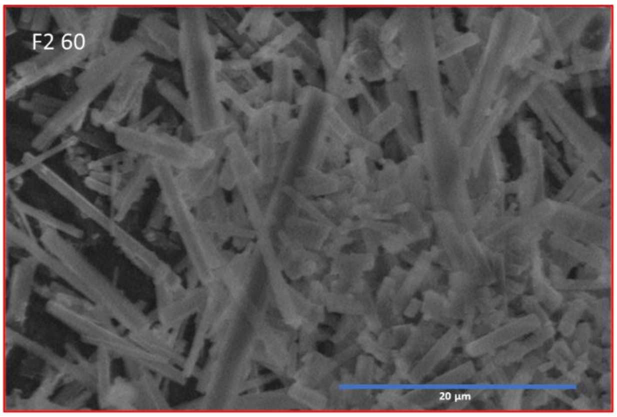
F2 60
20 µm

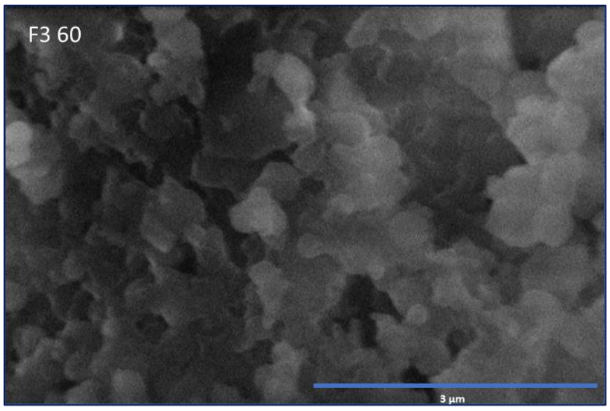
F3 60
3 µm

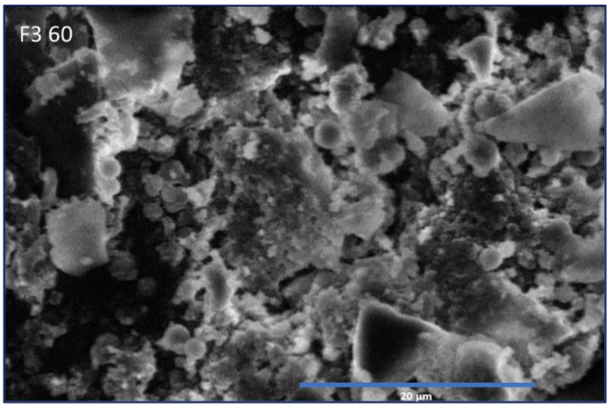
F3 60
20 µm

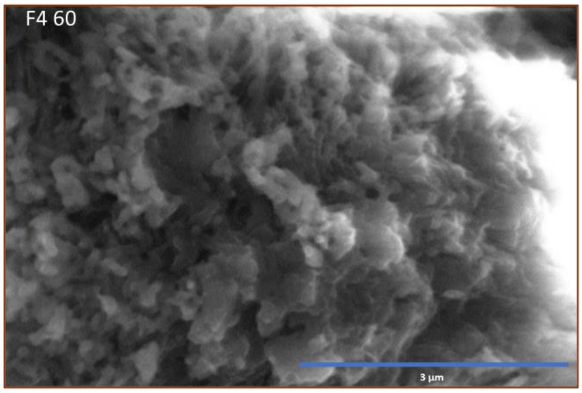
F4 60
3 µm

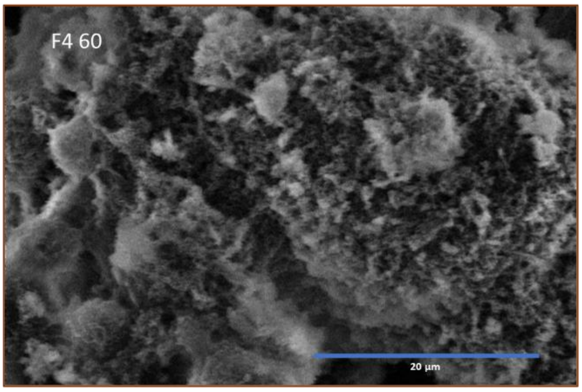
F4 60
20 µm

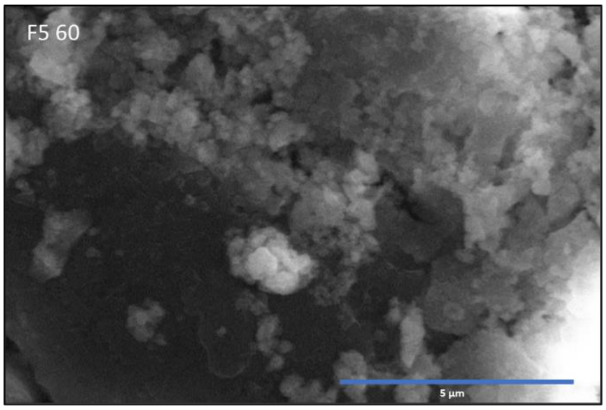
F5 60
5 µm

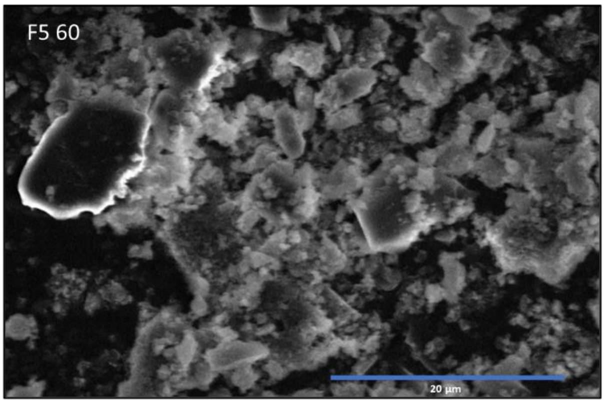
F5 60
20 µm

S.I.G/ TEM

Figure S.I.G/1 TEM images of the fractions from F1 to F5 for 20, 40 and 60 minutes of solvothermal treatment

TEM micrographs of the fractions for the synthesis at 60 min. doesn't show particle features specific or analogous to CDs. Structures or varying morphologies are observed. The fractions all display a kind of membrane or coating-like morphology that extend beyond the grid, in the space between corresponding to the holes of the grid. Such morphologies are likely the result of the aggregation of molecular fluorophore[4,5]. However, some nanoparticles can be observed in the fraction F_5. The size of this carbon particles is several tens of nanometers. This suggest that this carbon nanomaterial isn't carbon dots but rather a by-product significantly larger. It can explain the origin the signal signal of amorphous carbon powder we observed in powder x-ray diffraction and molecular fluorophores could be adsorbed to this by-product, endowing it with fluorescent properties. These relatively big carbon nanomaterials may also be responsible for the cloudy solution, to a certain extent, that we observe in the case of the fraction F_5. Overall, The fractions from F_1 to F_4 don't display the structures expected for carbon dots but rather appear as aggregated molecular fluorophores, a kind of supramolecular association of organic fluorophores. In addition to this, some relatively big carbon nanomaterial structures are observed in the fraction F_5.

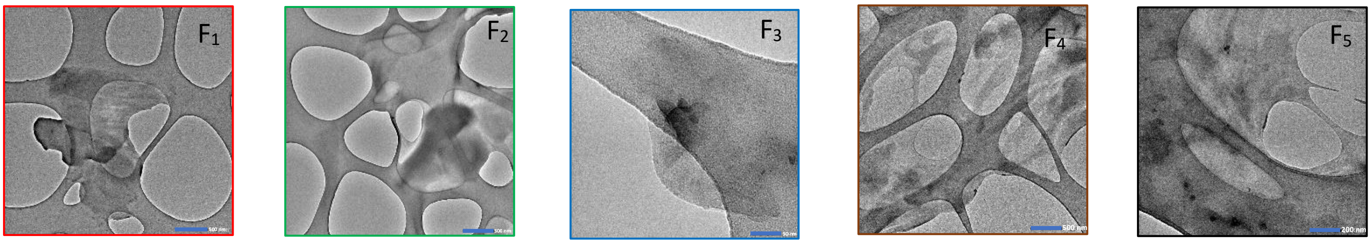

Optical properties

The UV-Vis spectra of the samples were recorded. For all the fractions, pronounced absorption is observed at around 300 nm, 400 nm and 500 nm. For a given fractions, the absorption spectra are fairly similar even when the duration of the synthesis varies. The F5 absorption spectra extend to slightly longer wavelength. Around 400 nm and 500 nm, significative absorption bands are observed. The absorption spectra of the fractions exhibit close shapes with the TNP absorption spectrum, in particular around 400 nm. It contrasts with the expected large UV-Vis absorption for carbon dots and rather suggests to some kind of molecular fluorophores derived from trinitropyrene. Emission spectra was also recorded. From F1 to F4, brightly luminescence can be observed under 365 nm UV, lamp. Luminescence slightly less intense. The fractions display similar emission around 600 nm. From F_1 to F_5, the maximum wavelength emission seems to shift slightly to the shorter wavelength, 618 nm for F1 to around 580, close to the TNP maximum wavelength emission spectrum.

S.I.H/ Ultraviolet-visible spectroscopy

Figure S.I.H/1 UV-visible absorbance spectra of TNP and the fractions obtained for durations of 20 and 40 minutes of solvothermal treatment

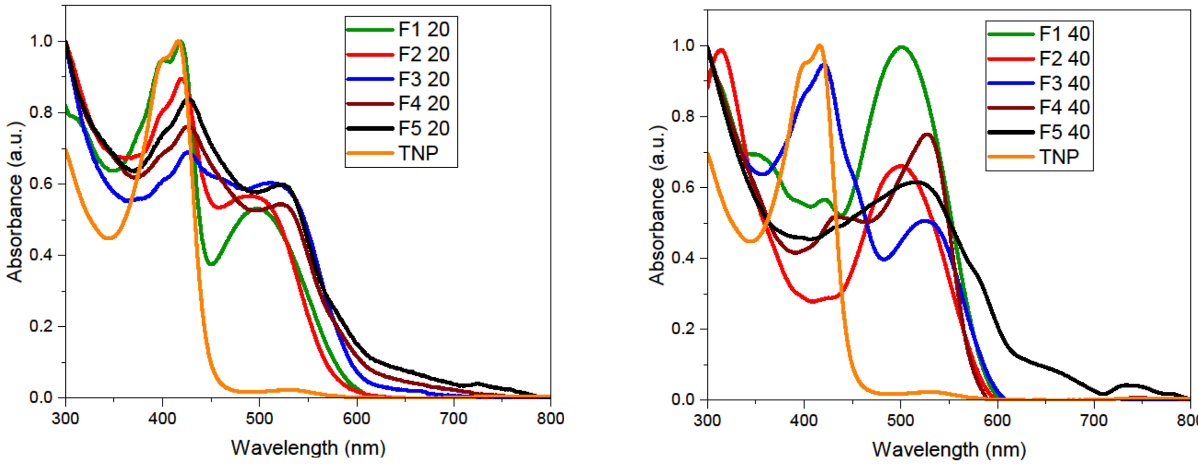

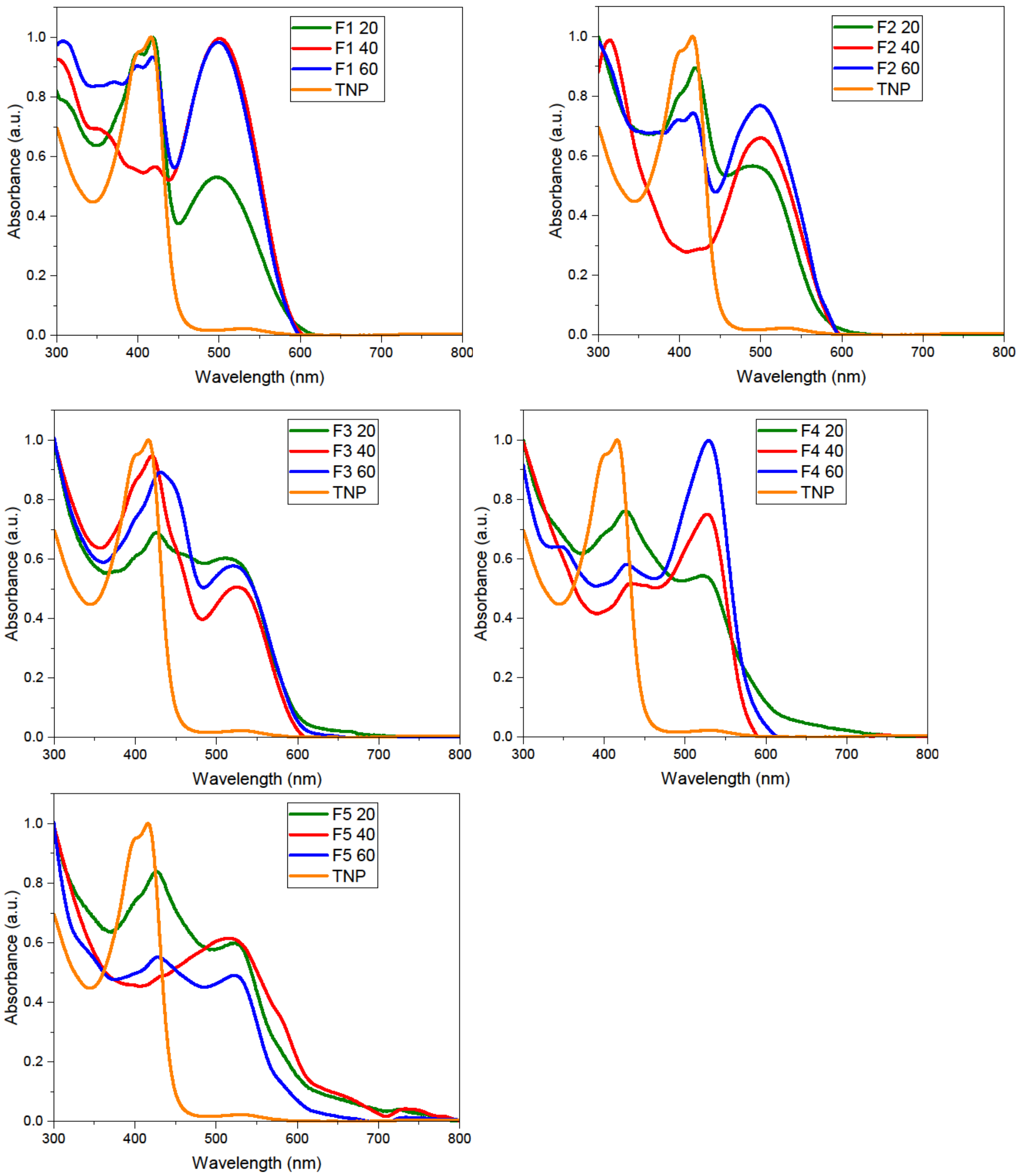
Absorbance (a.u.)
Wavelength (nm)
F1 20
F1 40
F1 60
TNP
F2 20
F2 40
F2 60
TNP
F3 20
F3 40
F3 60
TNP
F4 20
F4 40
F4 60
TNP
F5 20
F5 40
F5 60
TNP

S.I.J/ Fluorescence spectroscopy

Figure S.I.J/1 Florescence spectra of the fractions for durations of 20, 40 and 60 minutes of solvothermal treatment

The small peak around 730 nm for the TNP emission spectra is due to 2nd order diffraction but doesn't change the overall fluorescence of the TNP spectra.

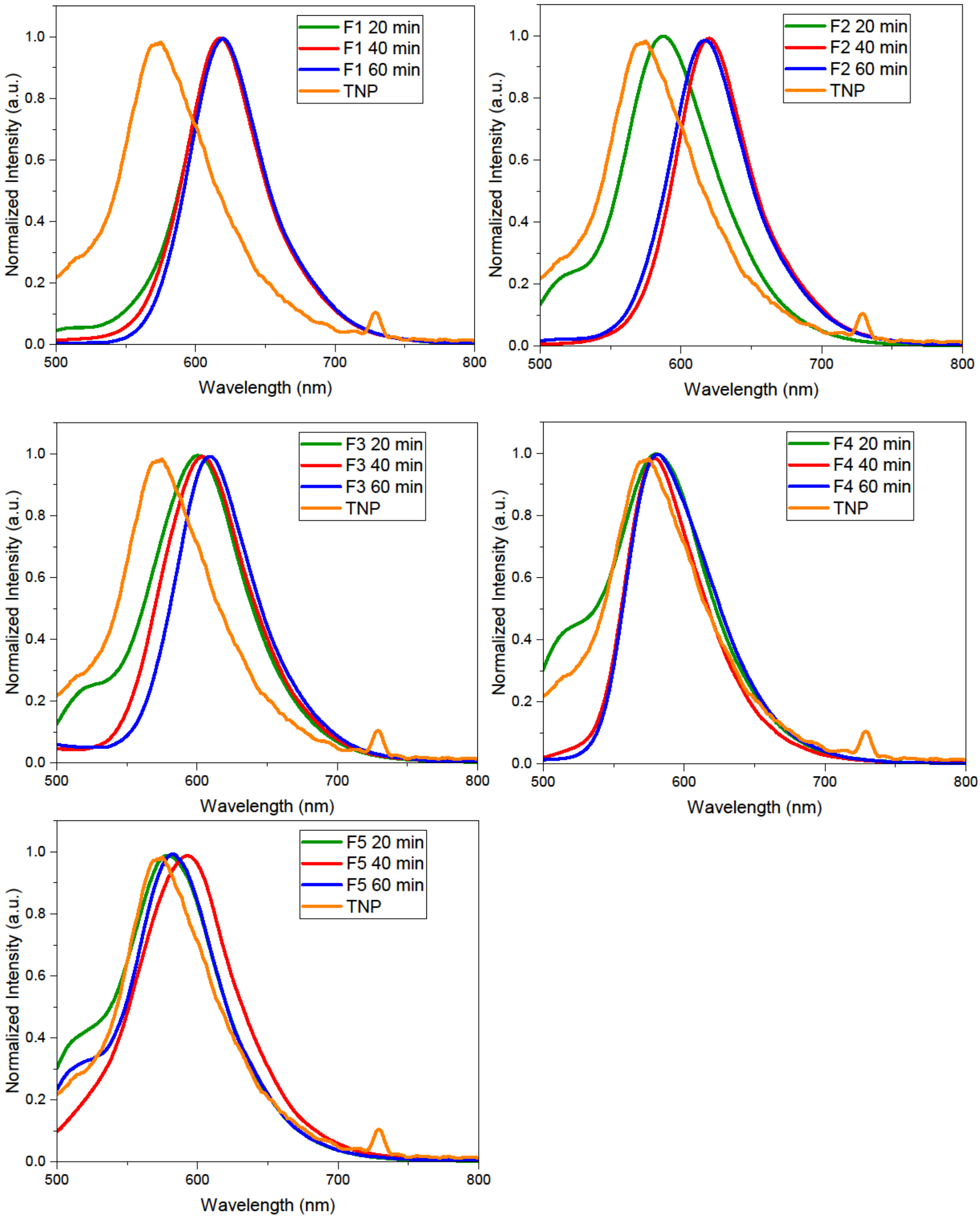

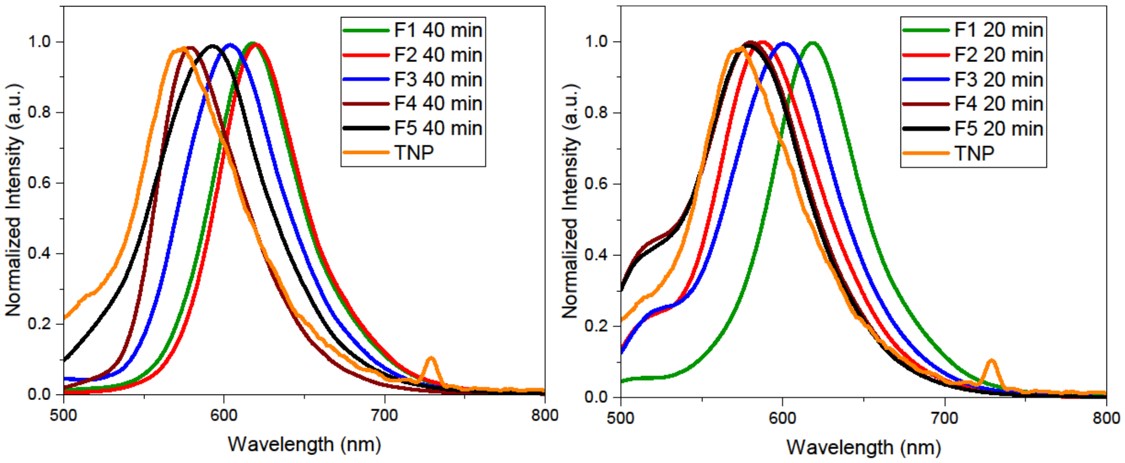

Table S.I.K/ Main structural information on the fractions F_1, F_2, F_3, F_4 and F_5 obtained for a 60 min microwave-assisted solvothermal treatment of TNP in DMF

		F1	F2	F3	F4	F5
**Techniques**	**XRD**	7.47°, 9.52°, 10.3°, 15,1° smaller peaks at 8.29°, 12.4° - rather crystalline phase	7.47°, 10°, 14,5°, 14,8° smaller peaks at 19.9°, 20.8°, 12.4° - rather crystalline phase	A few sharp peaks at low 2ϴ values and low intensity underlying large peaks around 10° and 20°	8.6°, 10.6°, 14.65°, 22.65°, 25.1°, 17.35°, smaller peaks at 11.26°, 12.45°, 19.63°, 21.69° - rather crystalline phase	Large peak at around 10° and 20°, rather amorphous phase
	**SEM**	Stacked thread-like structure of various size (µm size) and diameter	Rods structures of odd length (µm size)	Irregular sheets and sphere-shaped pieces	No distinct morphology	No distinct morphology
	**FTIR spectroscopy**	Peaks around 1516 cm^{-1} and 1346 cm^{-1} attributed to the asymmetric and symmetric stretching of the NO_2 bond, sp^3 C-H stretching (3100 cm^{-1}), C-H stretching (2920 cm^{-1}), C=C stretching (1590 cm^{-1}) and NO_2 shearing (860 cm^{-1})				
	**Raman spectroscopy**	1590 $cm^{-1}$and 1279 cm^{-1} can be attributed to NO2 asymmetric stretching and symmetric stretching, 1000-1300 cm^{-1} are associated to CH in-plane bending vibrations.				

1 M. E. Idrissi, S. J. Teat, P. F.-X. Corvini, M. J. Paterson, S. J. Dalgarno and P. Shahgaldian, *Chem. Commun.*, 2017, **53**, 1973–1976.
2 R. P. Bhatta, V. Kachwal, C. Climent, M. Joshi, P. Alemany, A. R. Choudhury and I. R. Laskar, *J. Mater. Chem. C*, 2023, **11**, 11399–11408.
3 Y.-F. Li, X. Wang, C.-Y. Wang, Y. Wang and Y.-W. Yang, *Tetrahedron*, 2023, **149**, 133718.
4 G. Batra, S. Sharma, K. Kaushik, C. Rao, P. Kumar, K. Kumar, S. Ghosh, D. Jariwala, E. A. Stach, A. Yadav and C. K. Nandi, *Nanoscale*, 2022, **14**, 3568–3578.
5 J. B. Essner, J. A. Kist, L. Polo-Parada and G. A. Baker, *Chem. Mater.*, 2018, **30**, 1878–1887.
